# Susceptibility loci revealed for bovine respiratory disease complex in pre-weaned holstein calves

**DOI:** 10.1186/1471-2164-15-1164

**Published:** 2014-12-22

**Authors:** Holly L Neibergs, Christopher M Seabury, Andrzej J Wojtowicz, Zeping Wang, Erik Scraggs, Jennifer N Kiser, Mahesh Neupane, James E Womack, Alison Van Eenennaam, Gerald Robert Hagevoort, Terry W Lehenbauer, Sharif Aly, Jessica Davis, Jeremy F Taylor, The Bovine Respiratory Disease Complex Coordinated Agricultural Project Research Team

**Affiliations:** Department of Animal Sciences, Washington State University, P.O. Box 646310, Pullman, WA 99164-6310 USA; Department of Veterinary Pathobiology, Texas A&M University, College Station, USA; Department of Animal Sciences, University of California Davis, Davis, USA; Extension Animal Sciences and Natural Resources Department, New Mexico State University, Las Cruces, USA; Department of Population Health and Reproduction, University of California Davis, Davis, USA; Division of Animal Sciences, University of Missouri, Columbia, USA

**Keywords:** Genomics, Bovine respiratory disease complex, Dairy, Calves, Loci

## Abstract

**Background:**

Bovine respiratory disease complex (BRDC) is an infectious disease of cattle that is caused by a combination of viral and/or bacterial pathogens. Selection for cattle with reduced susceptibility to respiratory disease would provide a permanent tool for reducing the prevalence of BRDC. The objective of this study was to identify BRDC susceptibility loci in pre-weaned Holstein calves as a prerequisite to using genetic improvement as a tool for decreasing the prevalence of BRDC. High density SNP genotyping with the Illumina BovineHD BeadChip was conducted on 1257 male and 757 female Holstein calves from California (CA), and 767 calves identified as female from New Mexico (NM). Of these, 1382 were classified as BRDC cases, and 1396 were classified as controls, with all phenotypes assigned using the McGuirk health scoring system. During the acquisition of blood for DNA isolation, two deep pharyngeal and one mid-nasal diagnostic swab were obtained from each calf for the identification of bacterial and viral pathogens. Genome-wide association analyses were conducted using four analytical approaches (EIGENSTRAT, EMMAX-GRM, GBLUP and FvR). The most strongly associated SNPs from each individual analysis were ranked and evaluated for concordance. The heritability of susceptibility to BRDC in pre-weaned Holstein calves was estimated.

**Results:**

The four statistical approaches produced highly concordant results for 373 top ranked SNPs that defined 126 chromosomal regions for the CA population. Similarly, in NM, 370 SNPs defined 138 genomic regions that were identified by all four approaches. When the two populations were combined (i.e., CA + NM) and analyzed, 324 SNPs defined 116 genomic regions that were associated with BRDC across all analytical methods. Heritability estimates for BRDC were 21% for both CA and NM as individual populations, but declined to 13% when the populations were combined.

**Conclusions:**

Four analytical approaches utilizing both single and multi-marker association methods revealed common genomic regions associated with BRDC susceptibility that can be further characterized and used for genomic selection. Moderate heritability estimates were observed for BRDC susceptibility in pre-weaned Holstein calves, thereby supporting the application of genomic selection to reduce the prevalence of BRDC in U.S. Holsteins.

**Electronic supplementary material:**

The online version of this article (doi:10.1186/1471-2164-15-1164) contains supplementary material, which is available to authorized users.

## Background

Bovine respiratory disease complex (BRDC) is the most common infectious disease and the leading natural cause of death among cattle in the United States [1], with the onset of disease generally considered to be initiated by a stressful event that suppresses the normal host immune response, thereby enabling opportunistic viral and bacterial infections of the respiratory tract. Many diverse pathogens can be involved in this process, such that bovine respiratory disease is considered to be etiologically complex (i.e., BRDC), and perhaps more challenging to control or mitigate than many diseases caused by a single, discrete pathogen. The most common viral pathogens associated with BRDC include: bovine herpesvirus 1, bovine parainfluenza virus 3, bovine viral diarrhea viruses 1 and 2, bovine respiratory syncytial virus, bovine adenoviruses A-D, and bovine coronavirus, whereas the most common bacterial pathogens are *Arcanobacterium pyogenes*, *Manheimia haemolytica, Pasteurella multocida, Histophilus somni,* and *Mycoplasma spp.*[2].

Bovine respiratory disease complex is commonly observed in beef and dairy calves, with both acute and long term effects on health and performance [3–5]. Acute effects include production or performance losses associated with the symptoms of BRDC that include fever, rapid breathing, repetitive coughing, nasal and/or eye discharge, diarrhea, dehydration and appetite depression [5, 6]. The prevalence of BRDC is temporally variable, with the proportion of affected feedlot cattle ranging from 5% to 44% over a 15 year period, with an average overall prevalence of 17% in U.S. feedlots [3, 7, 8]. In pre-weaned U.S. dairy calves, the prevalence of BRDC over a 20 year period ranged from 3.3 to 23.6% [4]. The mortality rate for BRDC in pre-weaned dairy calves was 22.5%, even though 93.4% of all heifers diagnosed with BRDC were treated with an antimicrobial agent, and the majority of these calves were also vaccinated for one or more known pathogens [4, 9]. Chronic effects of BRDC have resulted in reduced growth rates and decreased productivity of cows later in life [10, 11]. Cows affected by respiratory disease as a calf were twice as likely to die before calving, and calved at an older age as heifers, than those cows that were not affected by BRDC prior to 90 days of age [12, 13].

Despite the availability and use of vaccines for common respiratory pathogens, the prevalence of BRDC in dairy and beef animals is not declining [7, 14, 15]. Additional approaches to reduce the prevalence or prevent BRDC are needed to positively augment the use of vaccines and current best management practices aimed at reducing stress. Selection of breeding stock that are less susceptible to BRDC represents one viable approach that could be implemented, with the expectation of systematically reducing BRDC prevalence over the long term. Previous studies have identified genetic differences among animals in relation to BRDC susceptibility, thereby providing ample support and precedent for this hypothesis and investigation [16–19]. The objective of this study was to identify loci associated with BRDC susceptibility in pre-weaned Holstein calves via a case–control study design. Genome-wide association analyses (GWAA) identified genomic regions associated with BRDC that were largely concordant across four analytical approaches. This level of concordance has not often been reported in other Holstein GWAA studies that have evaluated multiple methods [20], which strongly underscores the potential for genomic selection enabled by BRDC susceptibility loci reported here. Additionally, heritability was estimated for BRDC susceptibility in both the individual and the combined CA and NM Holstein populations to further evaluate the potential for reducing the prevalence of BRDC through the selection of less susceptible breeding stock.

## Results and discussion

### Study animals

Prior to GWAA, seven of the pre-weaned California (CA) calves (n = 2021) were removed due to conflicts with their reported disease status and health scores (i.e., recording errors). Likewise, 73 (7.2%) of the control calves (48 males; 25 females) were later reclassified as BRDC cases prior to weaning (i.e., they developed clinical signs), and were subsequently removed from the analysis of the distribution of health scores (Additional file 1: Table S1). However, these animals did remain in the GWAA. Consequently, 2,014 calves (1,011 controls and 1,003 cases) from CA were used in this study. A total of 766 calves identified as females were enrolled from New Mexico (NM). Eighteen of the animals were subsequently identified to be a Jersey or a Jersey crossbred and were removed; leaving 748 Holstein identified heifers in the NM study; 372 as controls and 376 cases. Six percent of the heifers (n = 23) originally classified as matched controls later converted to BRDC case-status but remained in the study, as new diagnostic health scores and swabs were obtained at the time of conversion. Further details regarding the study animals (age, gender, proportion of males and females in the cases and control groups, and diagnostic health scores) may be found in Additional file 1: Table S1.

### Diagnostics

The observed pathogen profiles of the CA and NM animals differed for all pathogens with the exception of bovine herpes virus and bovine viral diarrhea virus, which were rarely detected (*P* < 0.05) (Table 1). The detection of different pathogens in CA and NM may have been influenced by the stage of disease at the time that the diagnostic swabs were obtained at each study site. The difference in the mean health scores between cases and controls (Additional file 1: Table S1) representing the two populations (i.e., CA vs NM; *P* < 0.001 in both populations) may simply reflect local differences in BRDC disease progression, possibly due to environmental differences or differences in BRDC pathogen(s) at the two study sites (Table 1). Notably, viruses that were important during the initiation of BRDC in Holstein calves may have been missed since the clinical manifestation of disease is necessary to identify cases using the McGuirk scoring system [6]. Moreover, some viruses are only rarely detected in the bovine pharynx beyond the first two days of infection (i.e., prior to their migration to the lung), which may have impacted our diagnostic results. Because this study occurred in an industry setting, the timing and certainty of pathogen exposure was unknown. However, the bacteriology and virology obtained from mid-nasal and deep pharyngeal swabs in this study collectively represent the largest ever dairy cattle diagnostic survey of respiratory pathogens which are likely to influence the clinical manifestation of BRDC. This survey is useful for a variety of reasons, including understanding why GWAA signals might potentially differ between locales, and for understanding the overall diversity in detectable pathogen profiles, as well as their putative frequency distributions among Holstein calves classified as BRDC cases and controls [6]. Therefore, the diagnostic data were helpful in providing a general overview of BRDC pathogen diversity at the time of phenotype classification (i.e., case vs control), but do not necessarily provide precise insight into the temporal and sequential progression of disease with associated pathogens across time. Nevertheless, the use of deep pharyngeal swabs has the very clear advantages of being a much more rapid, less costly, and less invasive sampling technique than transtracheal washes or bronchioalveolar lavage.

**Table 1:** **Pathogens identified from deep pharyngeal and mid-nasal swabs in Holstein calves from California and New Mexico**

Pathogen	*California n = 2,014	*^New Mexico n = 748	*California & ^New Mexico n = 2,763	^+^ Odds ratio	Odds ratio 95% confidence interval	Odds ratio ***P*** value
***Arcanobacterium pyogenes***	0.3 (0)	10.7 (4.3)	3.1 (1.2)	2.8	1.5-5.3	0.0003
***Histophilus somni***	1.7 (0.4)	3.2 (0.5)	2.1 (0.4)	4.9	2.0-14.6	<0.0001
***Manheimia haemolytica***	23.5 (11.1)	4.5 (3.5)	18.4 (9)	2.3	1.8-2.9	<0.0001
***Pasteurella multocida***	36.3 (23.6)	61.1 (54.8)	43.0 (32)	1.6	1.4-1.9	<0.0001
***Mycoplasma*** **spp.**	64.6 (57.1)	57.4 (48.7)	62.6 (54.8)	1.4	1.2-1.6	<0.0001
**Bovine corona virus**	9.6 (7.7)	50.0 (35.0)	19.9 (14.5)	1.5	1.2-1.8	0.0004
**Bovine respiratory syncytial virus**	20.8 (7.7)	4.9 (2.5)	16.3 (6.3)	2.9	2.2-3.8	<0.0001
**Bovine viral diarrhea virus**	0 (0)	1.3 (0)	0.4 (0)	NA	NA	NA
**Bovine herpes virus**	0 (0)	0 (0)	0 (0)	NA	NA	NA

*Percent of cases and controls (in parentheses) where individual pathogens were present in the pre-weaned Holstein calves. Animals classified as undetermined with respect to case–control status were not included in the summary statistics presented here.

^Jersey calves (n = 18) were removed;

^**+**^Odds ratio of being affected with BRDC when the pathogen was present when the animal was swabbed.

### Genome wide association analysis

*Heritability Estimates.* Implementation of EMMAX [21, 22] in conjunction with a genomic relationship matrix (EMMAX-GRM) [23] and an additive model produced moderate pseudo-heritability estimates (*h*^*2*^ = 0.21) for BRDC susceptibility in CA Holstein calves. The pseudo-heritability, as previously defined [21], is the proportion of phenotypic variance that is explained by the empirically estimated relationship matrix. Although it is not an exact analytical replicate of the heritability estimate produced by Restricted Maximum Likelihood Estimation (REML) in the Genomic Best Linear Unbiased Prediction (GBLUP) analyses, the EMMAX-GRM pseudo-heritability estimate was very similar to the GBLUP REML heritability estimate (*h*^2^ *=* 0.21) for BRDC susceptibility in the California calves. EMMAX-GRM analyses performed using an additive genetic model also produced a moderate pseudo-heritability estimate (*h*^2^ *=* 0.21) for BRDC susceptibility in the NM calves, which was nearly identical to the GBLUP REML estimate (*h*^2^ *=* 0.20). In a final analysis, we combined the NM and CA calves into a single case–control cohort. EMMAX-GRM pseudo-heritability for BRDC susceptibility within the combined cohort was again moderate (*h*^2^ *=* 0.13), but considerably lower than the estimates produced for the individual populations. This result was supported by the GBLUP REML estimated heritability for the combined population (*h*^2^ *=* 0.13), which was very similar to EMMAX-GRM (Table 2).

**Table 2:** **Heritability estimates of Holstein Calves from California, New Mexico and California and New Mexico combined populations**

Population	h ^2^	V _A_	V _E_
California-EMMAX-GRM	0.2110	0.0524	0.1959
California-GBLUP	0.2151	0.0535	0.1952
New Mexico-EMMAX-GRM	0.2139	0.0535	0.1966
New Mexico-GBLUP	0.2013	0.0501	0.1988
Combined-EMMAX-GRM	0.1318	0.0328	0.2161
Combined-GBLUP	0.1311	0.0326	0.2162

The heritability estimates of approximately 0.21 for BRDC susceptibility are greater than similar estimates for other health traits such as mastitis (0.04-0.05), cystic ovaries (0.05), ketosis (0.01-0.14), metritis (0.01-0.07) and lameness (0.02-0.03), but lower than estimates for displaced abomasum (0.32) and retained placenta (0.02-0.36) previously reported in dairy cows [24–26]. Bovine clinical mastitis (CM) is similar to BRDC in that a variety of microorganisms can be involved in the disease etiology. In CM, heritability estimates increase when the animal health phenotype is more precisely defined by the specific microorganism(s) involved in the CM etiology [27]. For example, heritability estimates for susceptibility to CM caused by Coliform species were found to be moderate (i.e., 0.17-0.19) [27], and similar to our estimates for BRDC, whereas heritability estimates were lower when CM cases were pooled (i.e., multiple, unrelated pathogens), or when CM cases were undetected [27–29], thereby underscoring the importance of precise animal disease phenotypes.

Moderate heritability estimates for BRDC susceptibility strongly suggest that there is ample opportunity to use genomic selection to reduce the prevalence of BRDC in Holstein dairy cattle. The reduction in heritability estimated for the combined CA and NM cohort, in comparison to the individual population estimates, is a complex phenomenon that likely reflects environmental differences as well as differences in the host genetic basis for differential susceptibility to a variety of BRDC pathogens that were detected at variable frequencies within the two populations. However, it should also be noted that the NM calves also tended to be older and presumably more clinically ill (as defined by the McGuirk Scoring System) [6] at sampling than did the CA calves, with the majority of the calves enrolled in the CA study being males, and nearly all of the calves enrolled in the NM study identified as being females by Illumina HD genotype (i.e., X and Y chromosome SNP calls; See Methods). Studies are currently underway to evaluate the statistical relationship between pathogen profiles, and the clinical scores (McGuirk Scoring System) [6] assigned to all Holstein calves.

### California population

**Figure 1:**
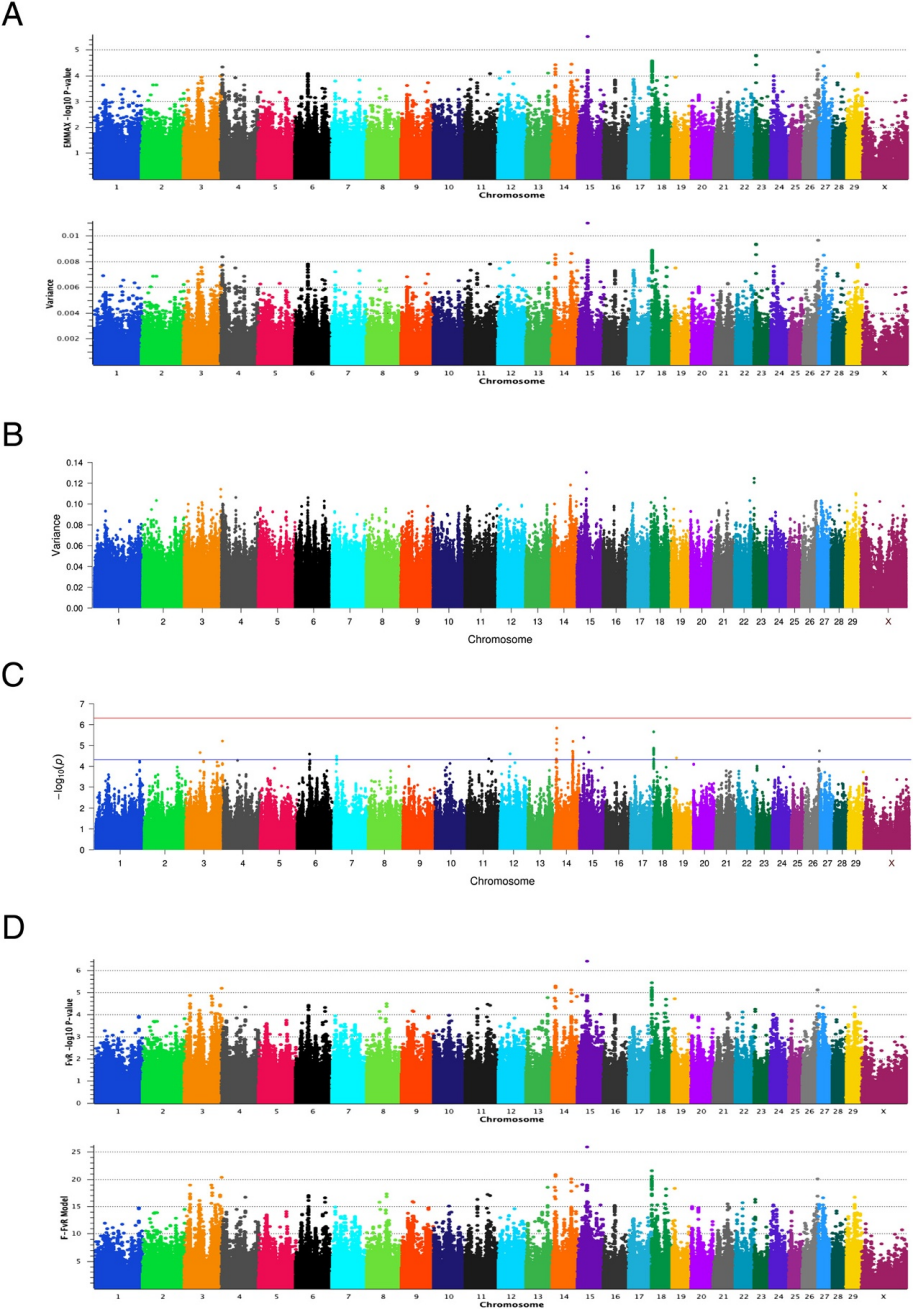
**Manhattan plots representing four genome wide association analyses for the binary BRDC case–control phenotype using (A) EMMAX-GRM, (B) GBLUP, (C) EIGENSTRAT and (D) Full versus reduced model regression (FvR) in the CA calves.** In **panel A**, the plot for EMMAX-GRM case–control with covariates of sex and age is shown. For EMMAX-GRM, the first panel shows the –log_10_ *P*-values on the Y axis and the second panel shows the proportion of variance explained by effects at a single SNP. In **panel B**, the plot for GBLUP case–control with covariates of sex and age is shown. The Y axis shows the proportion of variance explained by a sliding window of 7 SNPs centered on the third SNP within each window. In **panel C**, the plot for EIGENSTRAT case–control principal component corrected input data (top 100 principle components) with covariates of sex and age is shown. The –log_10_ *P*-values are indicated on the Y axis. The blue line indicates the Wellcome Trust threshold for SNPs with modest evidence for association with BRDC susceptibility, and the red line indicates the threshold for SNPs with strong evidence for association with BRDC susceptibility. In **panel D**, the Manhattan plot for FvR case–control principal component corrected input data (top 53 principal components) with covariates of sex and age is shown. The first FvR panel shows the –log_10_ *P*-values on the Y axis, and the second panel shows corresponding *F*-test values for the Full versus Reduced Model Analysis.

When the top 2,000 SNPs (i.e., largest effects) for CA were compared across the four analytical approaches for the case–control phenotype, a strong concordance of association with BRDC susceptibility was observed. Among the top 2,000 SNPs from each analytical approach, each SNP (or sliding window of 7 SNPs centered on the third SNP within each window for GBLUP) was given a ranking (i.e., 1 represented the most significant SNP or SNP window) and these rankings were summed across all analytical approaches for each population (Tables 3, 4 and 5). For the CA calves, Figure 1 shows the Manhattan plots for all analyses (EMMAX-GRM, FvR, GBLUP and EIGENSTRAT). In CA, the most significant locus across all four analyses was located on BTA15 between 30–31 Mb (Table 3). This was also the most significant locus detected using EMMAX-GRM (*P* = 2.95 × 10^-6^, Additional file 1: Table S2), GBLUP (proportion of additive genetic variance explained by the window of 7 SNPs or PVE = 0.13; note these windows slide one SNP at a time and so are not independent and the sum of PVE for all SNPs is greater than 1; Additional file 1: Table S3) and FvR (*P* = 3.74 × 10^-7^, Additional file 1: Table S4). The same BTA15 locus was also ranked 30^th^ among SNPs most strongly associated (*P* = 2.14 × 10^-5^) with BRDC susceptibility using EIGENSTRAT (Additional file 1: Table S5). This SNP association was supported by 18 adjacent SNPs ranked among the top 200 SNPs, and 38 SNPs ranked among the top 2,000 SNPs for either their magnitude of association or the proportion of variance explained with respect to BRDC susceptibility. The poliovirus receptor-related 1 (*PVRL1,* HGNC: 9706) gene is proximal and adjacent to this GWAA signal.

**Table 3:** **Concordance of genome-wide association analyses for the California calves with the loci most strongly associated with BRDC susceptibility for all analyses**

Chromosome (location Mb)	EMMAX-GRM ^+^	GBLUP ^+^	FvR ^+^	EIGENSTRAT ^+^	No. SNPs^	Lead SNP ^#^	Score*
BTA15 (30–31)	1	1	1	30	18	*rs41756356*	33
BTA14 (63–64)	14	7	15	6	34	*rs134331138*	42
BTA3 (119–120)	65	9	7	5	11	*rs41660991*	86
BTA23 (3–4)	5	2	76	115	5	*rs110158528*	198
BTA15 (14–15)	156	67	19	3	1	*rs136365736*	245
BTA19 (9–10)	73	170	37	44	1	*rs121919115*	324
BTA6 (42–43)	79	21	171	65	12	*rs43456007*	336
BTA4 (47–48)	74	19	192	63	1	*rs41651168*	348
BTA14 (11–12)	147	64	3	178	2	*rs133522438*	392
BTA6 (40–41)	131	72	172	32	12	*rs134070398*	412
BTA13 (71–72)	45	187	34	155	7	*rs110991341*	421
BTA7 (10–11)	97	274	135	41	6	*rs134195407*	547
BTA18 (0–1)	35	313	198	72	32	*rs109385868*	618
BTA3 (46–47)	178	364	178	31	2	*rs133069708*	678
BTA2 (45–46)	143	29	287	227	1	*rs136772856*	686
BTA11 (71–72)	304	375	51	48	1	*rs133739594*	778
BTA12 (6–7)	76	84	418	210	1	*rs135134119*	789
BTA3 (107–108)	294	315	98	110	1	*rs137396132*	817
BTA12 (33–34)	306	176	355	34	1	*rs133730139*	871
BTA16 (36–37)	125	140	112	529	13	*rs136788834*	906
BTA6 (95–96)	191	35	82	652	2	*rs41911495*	960

+Reflects the ranking of the best (lowest) ranked SNP for that genomic region for that analysis. Only scores <1000 are shown.

^Number of SNPs ranked among the top 200 SNPs (top 0.03%) that are found within this genomic region as being associated with BRDC susceptibility across the different analyses.

^#^The rs SNP identification number for the SNP with the lowest ranking for the QTL.

*Score is the sum of the ranks for the best ranked SNP for that genomic region across all analyses.

**Table 4:** **Concordance of genome-wide association analyses for the New Mexico calves with the loci most strongly associated with BRDC susceptibility for all analyses**

Chromosome (location Mb)	EMMAX-GRM ^+^	GBLUP ^+^	FvR ^+^	EIGENSTRAT ^+^	No. SNPs^	Lead SNP ^#^	Score*
BTA16 (70–71)	2	3	2	31	10	*rs41617348*	38
BTA14 (7–8)	5	48	30	7	17	*rs109009102*	90
BTA18 (65–66)	58	39	62	90	4	*rs135115567*	249
BTA12 (77–78)	62	30	175	14	1	*rs134174086*	281
BTA5 (23–24)	1	104	4	192	4	*rs109319628*	301
BTA13 (56–57)	38	19	184	61	7	*rs41630678*	302
BTA2 (2–3)	18	65	61	180	31	*rs134633034*	324
BTA13 (71–72)	112	94	116	38	11	*rs133881876*	360
BTA16 (64–65)	36	28	36	261	1	*rs42692646*	361
BTA28 (36–37)	84	23	204	89	2	*rs136647452*	400
BTA21 (47–48)	88	91	82	144	2	*rs134318902*	405
BTA25 (22–23)	40	22	98	273	6	*rs134095535*	433
BTA13 (67–68)	3	6	168	259	6	*rs41700634*	436
BTA10 (28–29)	145	75	74	167	6	*rs136924615*	461
BTA17 (72–73)	129	187	178	146	3	*rs134357791*	640
BTA14 (9–10)	124	179	237	159	2	*rs136578677*	699
BTA21 (50–51)	73	50	278	322	1	*rs133167991*	723
BTA19 (26–27)	128	38	81	590	3	*rs110385484*	837
BTA14 (10–11)	159	344	216	142	5	*rs110172337*	861
BTA8 (63–64)	194	161	15	491	14	*rs43557067*	861
BTA13 (6–7)	31	301	389	152	1	*rs135796451*	873
BTA13 (53–54)	44	82	403	342	13	*rs42388964*	882

+Reflects the ranking of the best (lowest) ranking SNP for that genomic region for that analysis. Only scores <1000 are shown.

^Number of SNPs ranked in the top 200 SNPs (top 0.03%) that are found in this genomic region as associated with BRDC susceptibility across the different analyses.

^#^The rs SNP identification number for the SNP with the lowest ranking for the QTL.

*Score is the sum of the ranking of the best ranking SNP for that genomic region across all analyses.

**Table 5:** **Concordance of genome-wide association analyses for the combined California and New Mexico calves with the loci most strongly associated with BRDC susceptibility for all analyses**

Chromosome (location Mb)	EMMAX-GRM ^+^	GBLUP ^+^	FvR ^+^	EIGENSTRAT ^+^	No. SNPs^	Lead SNP ^#^	Score*
BTA15 (30–31)	1	1	2	11	16	*rs41756356*	15
BTA19 (9–10)	40	188	20	1	1	*rs121919115*	249
BTA15 (31–32)	33	2	299	25	1	*rs109057462*	359
BTA11 (80–81)	123	103	71	64	9	*rs133204524*	361
BTA20 (0–1)	30	20	9	315	13	*rs110484875*	374
BTA3 (46–47)	55	192	91	81	2	*rs133069708*	419
BTA4 (47–48)	4	36	358	34	18	*rs136678678*	432
BTA17 (16–17)	26	3	49	397	3	*rs135082107*	477
BTA3(119–121)	41	339	118	82	3	*rs110977672*	580
BTA14 (11–12)	142	59	63	319	2	*rs133522438*	583
BTA17 (14–15)	80	135	26	391	20	*rs110488418*	632
BTA29 (48–49)	94	171	198	197	1	*rs136036800*	660
BTA4 (48–49)	21	209	455	3	7	*rs110213301*	688
BTA20 (0–1)	143	251	170	144	5	*rs110513803*	708
BTA16 (36–37)	162	280	224	92	14	*rs136059947*	758
BTA15 (14–15)	182	110	125	354	2	*rs136365736*	771
BTA3 (89–90)	342	146	138	147	2	*rs136571247*	773
BTA14 (81–82)	227	371	133	63	6	*rs132956286*	794
BTA18 (55–56)	107	26	647	26	1	*rs136121374*	806
BTA12 (58–59)	137	21	593	86	2	*rs135900256*	837
BTA7 (11–12)	297	207	4	378	1	*rs134912653*	886
BTA10 (1–2)	284	133	249	267	1	*rs136001993*	933
BTA17 (10–11)	105	480	100	260	2	*rs136672854*	945
BTA14 (62–63)	277	613	44	12	1	*rs136594328*	946
BTA20 (23–24)	60	202	461	254	9	*rs109301681*	977

+Reflects the ranking of the best (lowest) ranking SNP for that genomic region for that analysis. Only scores <1000 are shown.

^Number of SNPs ranked in the top 200 SNPs (top 0.03%) that are found in this genomic region as associated with BRDC susceptibility across the different analyses.

^#^The rs SNP identification number for the SNP with the lowest ranking for the QTL.

*Score is the sum of the ranking of the best ranking SNP for that genomic region across all analyses.

**Figure 2:**
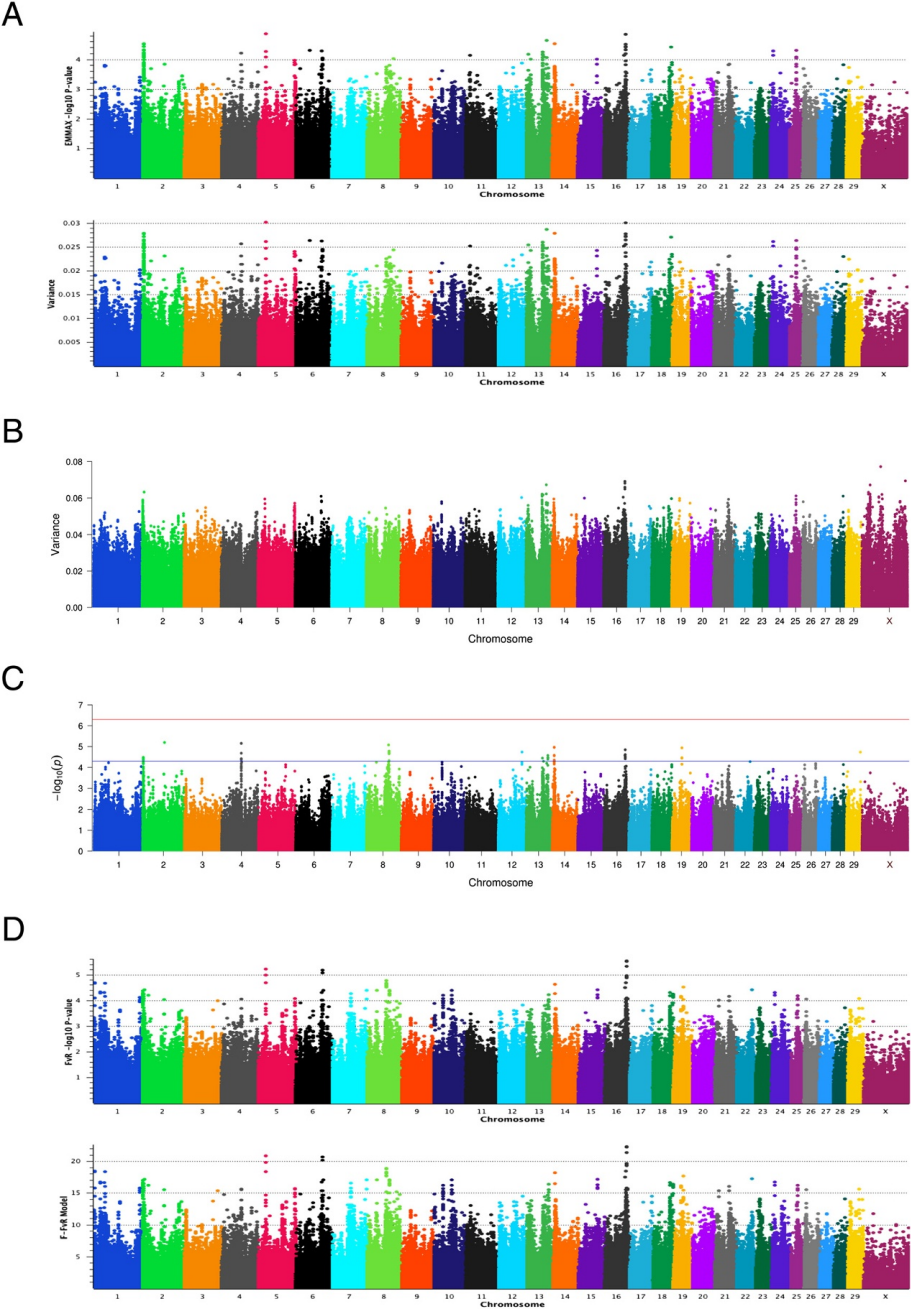
**Manhattan plots representing four genome wide association analyses for the binary BRDC case–control phenotype using (A) EMMAX-GRM, (B) GBLUP, (C) EIGENSTRAT and (D) Full versus reduced model regression (FvR) in NM calves.** In **panel A**, the plot for EMMAX-GRM case–control with covariates of sex and age is shown. For EMMAX-GRM, the first panel shows the –log_10_ *P*-values on the Y axis and the second panel shows the proportion of variance explained by effects at a single SNP. In **panel B**, the plot for GBLUP case–control with covariates of sex and age is shown. The Y axis shows the proportion of variance explained by a sliding window of 7 SNPs centered on the third SNP within each window. In **panel C**, the plot for EIGENSTRAT case–control principle component corrected input data (top 5 principal components) with the covariate age is shown. In **panel C**, the –log_10_ *P*-values are on the Y axis. The blue line indicates the Wellcome Trust threshold for SNPs with modest evidence for association with BRDC susceptibility, and the red line indicates the threshold for SNPs with strong evidence for association with BRDC susceptibility. In **panel D**, the Manhattan plot for FvR case–control principal component corrected input data (top 9 principal components) with covariates of sex and age is shown. The first FvR panel shows the –log_10_ *P*-values on the Y axis, and the second panel shows corresponding *F*-test values for the Full versus Reduced Model Analysis.

*PVRL1* is a member of the immunoglobulin superfamily that is known to mediate entry of several herpes viruses, including bovine herpes virus 1 (*BHV-1*), the causative agent of infectious bovine rhinotracheitis (IBR), which is also a known etiological agent of BRDC [30]. Notably, *BHV-1* was not diagnostically detected in the mid-nasal or pharyngeal swabs from the CA Holstein calves, but previous experimental challenge and pathology studies have demonstrated that many respiratory viruses, including *BHV-1,* are only transiently present in the pharynx prior to settling into the lung (Laurel Gershwin, personal communication). Therefore, it is unsurprising that *BHV-1* was not detected by our diagnostic procedures, although the host genomic signature of this pathogen was clearly detected and well supported statistically by all GWAA approaches. Our failure to detect *BHV-1* likely reflects the temporal and/or transient differences in viral expression and pathology (i.e., since the swabs were taken only at one time point), including latency of the virus in the trigeminal ganglion, or perhaps other factors, both known and unknown [31]. *BHV-1* causes a lifelong latent infection and sporadic shedding of the virus which can lead to horizontal transmission to other animals in the herd. In addition to BRDC, IBR also has the potential to cause rhinotracheitis, vaginitis, balanoposhitis, abortion, conjunctivitis, and enteritis [32]. Ulcers of *BHV-1* infected animals often occur in the mouth and nose, with mortality as high as 10% [33].

A second locus of interest identified across all analyses was located on BTA14 between 63–64 Mb. It contained the 6^th^ ranked SNP by EIGENSTRAT (*P* = 6.47 × 10^-6^, Additional file 1: Table S5), the 7^th^ ranked SNP by GBLUP (PVE = 0.12, Additional file 1: Table S3), the 14^th^ ranked SNP by EMMAX-GRM (*P* < 3.55 × 10^-5^, Additional file 1: Table S2), and the 15^th^ ranked FvR SNP (*P* = 1.09 × 10^-5^, Additional file 1: Table S4). This locus was supported by 34 top ranked SNPs, and was located near positional candidate genes *BAALC*, *ATP6V1C1* and *AZIN1*.

The most highly ranked SNP in this region across all analyses was proximal to *AZIN1* (antizyme inhibitor 1, HGNC: 16432), a gene that is involved in many biological processes, and that is most highly expressed in both lung and skeletal muscle [34]. *AZIN1* has been associated with Fanconi’s anemia, tumorogenesis, cirrhosis of the liver, and regulation of polyamine synthesis [35–37]. The *AZIN1* protein (Az1) regulates ornithine decarboxylase stability, which degrades polyamines, and in turn, up-regulates *AZIN1*. Interestingly, ornithine carboxylase activity and elevated polyamine levels are detected when cells are transformed by viral agents [38]. Individuals with deleterious mutations involving *AZIN1* (i.e., qualitative and/or quantitative) may be more likely to become infected with viruses, such as hepatitis C [38]. Some mutations in *AZIN1* may also enhance susceptibility to other viruses, such as those implicated in the clinical manifestation of BRDC. Flanking genes within this region include brain and acute leukemia cytoplasmic (*BAALC, HGNC:* 14333), and ATPase, H+ transporting, lysosomal 42 kDa, V1 subunit C1 (*ATP6V1C1, HGNC:* 856). Mutations in *BAALC* are implicated in myeloid leukemogenesis, and are associated with a poor prognosis in acute myeloid leukemia as well as acute lymphoblastic leukemia [39]. The ATPases are proton pumps that are involved in a wide variety of physiological processes [40]. In drosophila, the overexpression of V1 subunits was recently found to alter the replication capacity of influenza virus [41]. It is also conceivable that alteration of V1 subunits could potentially play a role in the replication capacity of other viruses, such as those involved in BRDC [41].

The third most significant locus across all analyses for CA was a 91 kb region defined by 11 SNPs on BTA3 between 119 and 120 Mb. The SNP rankings for EIGENSTRAT, FvR and GBLUP were very similar (i.e., 5^th^, 7^th^, and 9^th^, respectively), while EMMAX-GRM ranked this locus 65^th^ (Table 3**;** Additional file 1: Table S3, Additional file 1: Table S4, and Additional file 1: Table S5). No annotated genes are known to reside in this region of the genome.

The fourth most significant locus for CA across all analyses was a 10 kb region on BTA23 between 3 and 4 Mb (Table 3). This was the 2^nd^ most highly ranked locus for GBLUP (PVE = 0.13, Additional file 1: Table S3), 5^th^ for EMMAX-GRM (*P* = 1.65 × 10^-5^, Additional file 1: Table S2), and the 76^th^ and 115^th^ ranked marker for FvR (*P* = 5.58 × 10^-5^) and EIGENSTRAT (*P* = 1 × 10^-4^), respectively. The BTA23 SNPs identified as being associated with BRDC susceptibility are within an intron and exon of the dystonin gene (*DST* or *BPAG1, HGNC: 1090*). Interestingly, the previously described positional candidate gene *PVRL1* and *DST* share similar functions, as they both play a role in herpes virus infections [42, 43], one of the common BRDC pathogens responsible for IBR.

The dystonin protein is essential for anchoring epidermal cells to the underlying basement membrane, and dystonin proteins also function as autoantigens in a group of skin diseases that affect humans [44, 45]. Dystonin is also an integral component of hemidesmosomes, and is regulated by interferon gamma [46]. Relevant to our BRDC GWAA, dystonin is important in the transport of herpes simplex virus 1 capsids to the nucleus of the host cell through the microtubule network [42, 43]. Moreover, if the dystonin protein is depleted, capsid transport and efficient herpes virus infections are strongly inhibited [42, 43].

The locus ranked fifth across all GWAA approaches in the CA population was on BTA15 between 14–15 Mb (Table 3). This locus was ranked 3^rd^ (*P* = 4.28 × 10^-6^) by EIGENSTRAT (Additional file 1: Table S5), 19^th^ by the FvR approach (*P* = 1.31 × 10^-5^, Additional file 1: Table S4), 67^th^ by GBLUP (PVE = 0.10), and 156^th^ by EMMAX-GRM (*P* = 0.003). The associated SNP lies within intron two of mastermind-like 2 (*MAML2)* which has been associated with mucoepidermoid carcinoma, and height in humans [47–49].

### New Mexico population

Manhattan plots for the NM calves for all analyses are shown in Figure 2. The most highly ranked locus across all GWAA approaches for NM was located on BTA16 between 70 and 71 Mb (Table 4). This locus was defined by 10 SNPs and contained the second most highly ranked SNP for FvR (*P* = 2.93 × 10^-6^, Additional file 1: Table S4), the second highest ranked SNP for EMMAX-GRM (*P* = 1.39 × 10^-5^, Additional file 1: Table S2), the third highest ranked SNP for GBLUP (PVE = 0.07, Additional file 1: Table S3) and the 31^st^ ranked SNP for EIGENSTRAT (*P* = 2.99 × 10^-5^). Investigation of the associated region (105 kb) revealed two uncharacterized loci (*LOC101905027* and *LOC101904971;* Entrez gene: 101905027 and 101904971) [50], and no annotated genes.

Across all GWAA analyses, the second most highly ranked locus for NM was located on BTA14 between 7 and 8 Mb and was characterized by 17 SNPs (Table 4). The 25 kb region was located 2 and 9 kb away from the transfer RNA glycine (anticodon CCC) (*TRNAG-CCC,* Entrez gene: 100126533) and transfer RNA cysteine (anticodon ACA) (*TRNAC-ACA,* Entrez gene: 100499315) genes, respectively. This region contained the 5^th^ ranked SNP for EMMAX-GRM (*P* = 2.97 × 10^-5^, Additional file 1: Table S2), the 7^th^ ranked SNP for EIGENSTRAT (*P* = 1.09 × 10^-5^, Additional file 1: Table S5), the 30^th^ ranked SNP for FvR (*P* = 2.28 × 10^-5^, Additional file 1: Table S4), and the 48^th^ ranked SNP for GBLUP (PVE = 0.06).

The third ranked QTL for NM was on BTA18 (65–66 Mb) (Table 4). This region contained the 39^th^ ranked SNP for GBLUP (PVE = 0.06), the 58^th^ ranked SNP for EMMAX-GRM (*P* = 0.001), the 62^nd^ ranked SNP for FvR (*P* = 5.69 × 10^-5^) and the 90^th^ ranked SNP for EIGENSTRAT (*P* = 7.4 × 10^-5^). This QTL spanning 7.2 kb involved 4 SNPs located in introns 1–3 of the zinc finger protein 850 (*ZNF850,* HGNC: 27994) gene. At present, little is known about the function of this gene and protein, which precludes specific biological inferences in relation to BRDC.

The 4^th^ ranked locus for NM was located on BTA12 (77–78 Mb) (Table 4). This locus was only represented by a single SNP, and was most highly ranked by EIGENSTRAT (ranked 14^th^, *P* = 1.82 × 10^-5^, Additional file 1: Table S5). This locus was ranked 30^th^ by GBLUP (PVE = 0.06, Additional file 1: Table S3), 62^nd^ by EMMAX-GRM (*P* = 0.0001), and 175^th^ by FvR (*P* = 0.0002). Heparan sulfate 6-O-sulfotransferase 3 (*HS6ST3, HGNC:* 19134) is the only annotated gene in this region. One previous study suggests that *HS6ST3* may perform diverse functions [51], and more recent studies have associated *HS6ST3* with obesity in humans [52, 53].

The 5^th^ ranked locus for NM was located on BTA5, between 23 and 24 Mb (Table 4). This locus, spanning 3.6 kb, had the highest ranking SNP for EMMAX-GRM (*P* = 1.22 × 10^-5^, Additional file 1: Table S2), ranked 4^th^ with the FvR approach (*P* = 6.04 × 10^-6^, Additional file 1: Table S4), ranked 104^th^ with GBLUP (PVE 0.06), and ranked 192^nd^ with EIGENSTRAT (*P* = 0.0002). No genes or obvious biological candidates were found in this genomic region, although an expressed sequence tagged site (EST *Bt.86019*) [54] detected in bovine kidney tissue was found to underlie this peak.

### Combined California and New Mexico population

**Figure 3:**
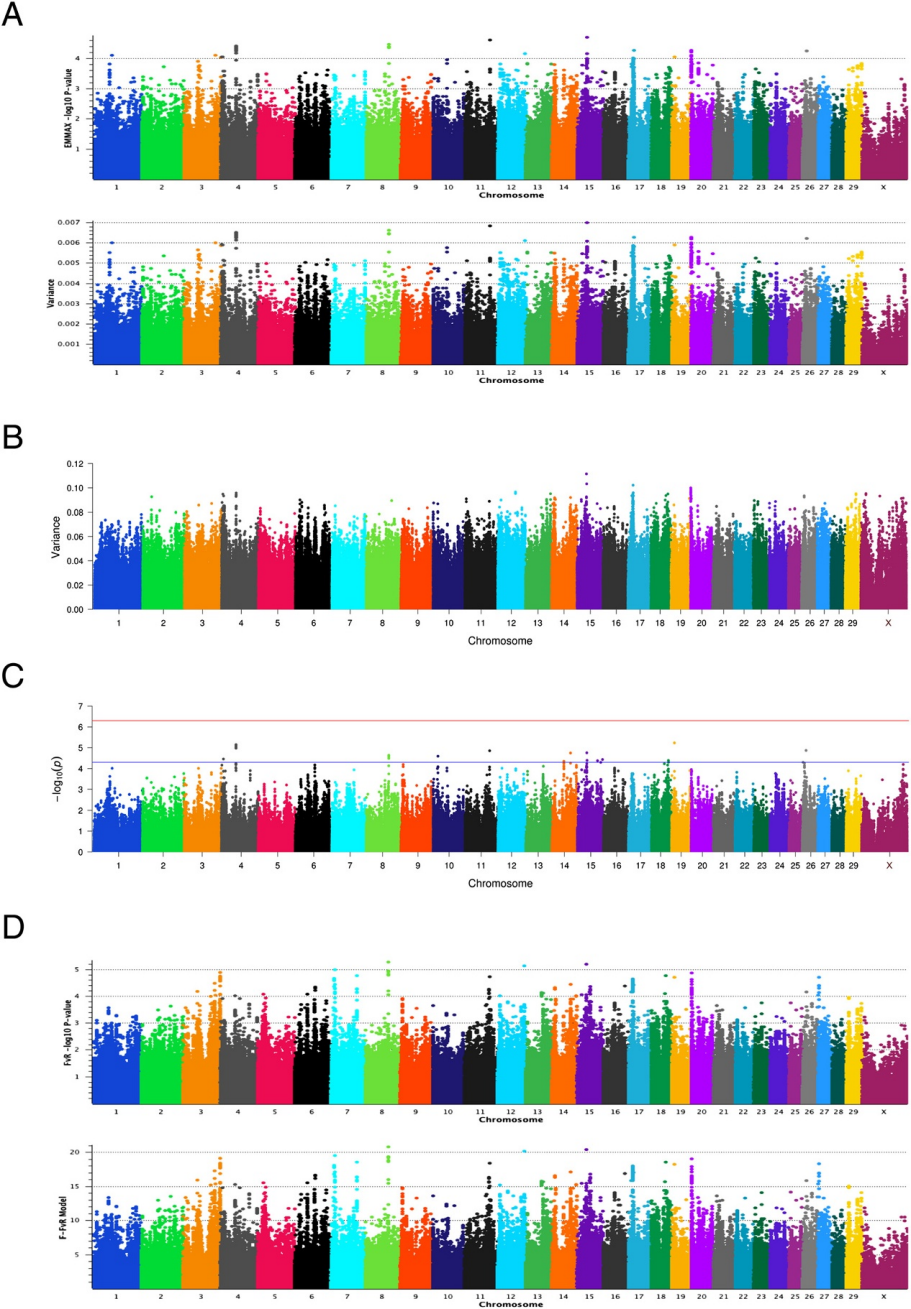
**Manhattan plots representing four genome wide association analyses for the binary BRDC case–control phenotype using (A) EMMAX-GRM, (B) GBLUP, (C) EIGENSTRAT and (D) Full versus reduced model regression (FvR) in the CA and NM calves combined.** In **panel A**, the plot for EMMAX-GRM case–control with covariates of sex, age, and herd of origin is shown. For EMMAX-GRM, the first panel shows the –log_10_ *P*-values on the Y axis and the second panel shows the proportion of variance explained by effects at a single SNP. In **panel B**, the plot for GBLUP case–control with covariates of sex and age is shown. The Y axis shows the proportion of variance explained by a sliding window of 7 SNPs centered on the third SNP within each window. In **panel C**, the plot for EIGENSTRAT case–control principle component corrected input data (top 80 principal components) with covariates of sex and age is shown. The –log_10_ *P*-values are indicated on the Y axis. The blue line indicates the Wellcome Trust threshold for SNPs with modest evidence for association with BRDC susceptibility, and the red line indicates the threshold for SNPs with strong evidence for association with BRDC susceptibility. In **panel D**, the Manhattan plot for FvR case–control principal component corrected input data (top 91 principal components) with covariates of sex, age, and herd of origin is shown. The first FvR panel shows the –log_10_ *P*-values on the Y axis, and the second panel shows corresponding *F*-test values for the Full versus Reduced Model Analysis.

When the top 2,000 SNPs (i.e., largest effects) for the combined population (CA + NM Holstein calves) were compared across the four analytical approaches for the case–control phenotype, we noted that the results were skewed toward those initially reported for the CA population (Table 3, Figure 1, Figure 3, Additional file 1: Table S2, Additional file 1: Table S3, Additional file 1: Table S4, Additional file 1: Table S5). This was relatively unsurprising, as the CA Holstein population had nearly three times the number of study animals as did the NM population, with the top ranked locus for BRDC susceptibility in the combined population being the same top ranked locus identified for the CA Holsteins (i.e., positional candidate *PVRL1*, see Table 3). Underlying this QTL signal was the highest ranked SNP using EMMAX-GRM (*P* = 1.95 × 10^-5^, Additional file 1: Table S2) and GBLUP (PVE = 0.11, Additional file 1: Table S3), the second highest ranked SNP for FvR (*P* = 6.42 × 10^-6^, Additional file 1: Table S4), and eleventh ranked SNP for EIGENSTRAT *(P* = 1.75 × 10^-5^, Additional file 1: Table S5).

Across all analyses, the second strongest association (n = 1 SNP) in the combined cohort was observed on BTA19 (9–10 Mb) (Table 5). This SNP was the highest ranked by EIGENSTRAT (*P* = 5.82 × 10^-6^, Additional file 1: Table S5), but was also highly ranked by the FvR approach (Rank = 20^th^, *P* = 1.97 × 10^-5^, Additional file 1: Table S4), and by EMMAX-GRM (Rank = 40^th^, *P* = 8.91 × 10^-5^). This SNP was ranked 188^th^ by GBLUP (PVE = 0.09). The BTA19 SNP associated with BRDC susceptibility is located within intron 7 of the myeloperoxidase (*MPO, HGNC:* 7218) gene. The protein encoded by *MPO* is central to the activity of neutrophils, and sputum myeloperoxidase is recognized as a putative biomarker for neutrophil activity in pulmonary disease [55].

For the combined cohorts (CA and NM), the third highest ranked locus across all analyses was represented by a single SNP located on BTA15 (31–32 Mb) (Table 5). This SNP was the second highest ranked SNP by GPLUP (PVE = 0.10, Additional file 1: Table S3), the 25^th^ ranked SNP by EIGENSTRAT (*P* = 4.04 × 10^-5^, Additional file 1: Table S5), the 33^rd^ ranked SNP by EMMAX-GRM (*P* = 6.9 × 10^-5^), and the 299^th^ ranked SNP by FvR (*P* = 0.003). At present, no annotated genes have been described within 50 kb of this SNP, thereby precluding the identification of one or more suitable positional candidate genes.

The fourth ranked genomic region for the combined cohort (CA + NM) was located on BTA11 (80–81 Mb), and was ranked 64^th^ among the results obtained using EIGENSTRAT (*P* = 8.42 × 10^-5^, Table 5), 71^st^ using FvR (*P* = 5.33 × 10^-5^), 103^rd^ using GBLUP (PVE = 0.09), and 123^rd^ using EMMAX-GRM (*P* = 0.0002). The most proximal gene to this SNP is retinol dehydrogenease 14 (all-trans/9-cis/11-cis) (*RDH14,* HGNC: 19979), which is located approximately 13 kb away. This gene encodes a mitochondrial protein that recognizes retinoids as substrates and may protect the mitochondria from oxidative stress associated with exposure to retinaldehyde [56].

The fifth ranked locus identified by all methodological approaches revealed a strong positional candidate gene on BTA20 (0–1 Mb) that is supported by 13 SNPs and spans 19 kb (Table 5). This QTL was identified by SNPs among the top 30 ranked for FvR (ranked 9^th^, *P* = 1.32 × 10^-5^ Additional file 1: Table S4), GBLUP (ranked 20^th^, PVE = 0.10; Additional file 1: Table S3) and EMMAX-GRM (ranked 30^th^, *P* = 6.02 × 10^-5^, Additional file 1: Table S2). This SNP was ranked 315^th^ by EIGENSTRAT (*P* = 0.0004). The most strongly associated SNPs were located within introns 2 and 10 of the slit homolog 3 (*SLIT3, HGNC: 11807*)) gene [57]. *SLIT3* is a very large gene spanning 727 kb*.* The protein encoded by *SLIT3* has numerous functions, one of which includes the down regulation of inflammatory mediators such as IL-1β, Il-6 and IL-8 [58]. *SLIT3* has also been implicated in cell migration, axon guidance, inflammation, angiogenesis and organogenesis [59]. The *SLIT* family of proteins features a tandem of four leucine-rich repeat domains which mediate binding to the immunoglobulin-like domains of the Roundabout (Robo) family of genes [60]. SLIT-Robo signaling has been implicated in immune response through the regulation of dendritic cell migration [61]. Airway dendritic cells are key regulators of pulmonary immune responses, and in the human lung, dendritic cells continuously present antigenic information from the airways to the pulmonary lymph nodes, thereby modulating the regulation of immune responses [62]. Therefore, perturbations of bovine *SLIT3* function may also influence susceptibility to BRDC, making it a plausible functional candidate gene for future investigations.

## Conclusions

Differences existed in the CA and NM Holstein calf populations in the ages of the calves when sampled, the average McGuirk health score [6] of the cases and controls, and the gender of the calves. These differences were incorporated into the analysis models to account for age and gender of the calves. The differences between the CA and NM pathogens detected at sampling likely had an impact on the BRDC susceptibility loci detected in the calves.

The BRDC heritability estimate of 0.21 for CA and NM calves was consistent across the two populations, but dropped to 0.13 when the populations were combined. This decline is most likely due to pathogen and environmental differences at the two study sites. However, the moderate heritability (0.13) estimated for BRDC susceptibility in the combined Holstein population is sufficient to support a nation-wide approach to reduce the prevalence of BRDC via genomic selection. Although each of the methods used to conduct the GWAA have strengths and weaknesses, the concordance between the loci associated with BRDC susceptibility across the four approaches has resulted in the identification of loci that are strong candidates for harboring causal mutations, and these QTL signals are less likely to have arisen solely by type 1 error. Several biologically meaningful positional candidate loci were identified in this study. Among these positional candidates are genes on BTA15 (*PVRL1*) and BTA23 (*DST*) that are known to mediate herpes virus entry into host cells, a gene involved in viral susceptibility on BTA14 (*AZIN1*), and a gene that modulates inflammation on BTA20 (*SLIT3*). Studies in *Bos taurus* feedlot cattle are currently underway to determine if some of the same loci are associated with enhanced susceptibility to BRDC in older cattle from different breeds.

Selection and breeding of less susceptible cattle with respect to BRDC is the most sustainable approach to reducing the prevalence of disease, as it will decrease the use of therapeutic antibiotics, lead to superior production efficiency, require less handling, and ultimately be more profitable. Studies are currently underway to identify the causal mutations associated with enhanced BRDC susceptibility, as an alternative to relying on array-based SNP markers as imperfect predictors of these mutations. Moreover, unlike approaches that rely on markers in linkage disequilibrium (LD) with the causal mutations for genomic selection, the accuracy of utilizing causal mutations to predict susceptibility will not decay over time, and likely will have utility across breeds, which enhances the value and longevity of the final marker panel to the industry. Identification of the causal mutations underlying BRDC QTL will ensure that the initial work of this study is sustainably translated to industry, with QTLs associated with enhanced BRDC susceptibility providing the foundation for identifying causal mutations with the largest effects on susceptibility, so that these may be incorporated into predicted transmitting abilities (PTA) for commercial dairy artificial insemination (AI) sires. As greater than 70% of the dairy calves in the U.S. are conceived by AI annually [63] this will provide immediate translation of our discoveries to a large segment of the U.S. dairy industry.

## Methods

### Study population

This study was conducted with an approved animal use protocol from the Institutional Animal Care and Use Committees at the University of California at Davis (16431), Washington State University (04110), New Mexico State University (2011–010), Texas A&M University (2014–0032) and the University of Missouri (7505). California Holstein calves were enrolled in a case–control study from July 2011 through January 2012. Collectively, 2,014 calves were enrolled as cases (n = 1,003) and controls (n = 1,011), with 1,257 males and 757 females. Animals in New Mexico were enrolled from August 2011 through June 2012 in a case control study at three calf raising facilities within the Clovis and Portales areas of New Mexico. All study animals were identified as heifers in the field, and consisted of 372 controls and 376 cases. The animals were sampled such that the proportions of cases and controls were almost identical across seasons of the year and locations in order to eliminate these effects as sources of variation in the experimental design. Details on the calf raising facilities, and the management and vaccination of the calves are specified in Additional file 1.

### BRD and control calf determination

Calves treated with antibiotics within 10 days prior to study onset were not considered for enrollment since treatment could facilitate negative bacterial cultures. Calves younger than 22 days of age were not enrolled to avoid potential false positive results during viral diagnostic tests (i.e., real time quantitative PCR, qPCR) due to the modified-live vaccines administered at 1 and 8 days of age [64]. Calves housed in hutches between the ages of 27 and 60 days of age were closely monitored and considered for enrollment as either BRDC cases or controls, based on the assignment of calf health scores using a respiratory screening tool developed by McGuirk [6]. Calves were observed before feeding (in the early morning) and assigned a health score as follows: 0 for normal, 1 for slightly abnormal, 2 for abnormal, and 3 for severely abnormal, with a numeric score also assigned for rectal temperature, cough, nasal discharge, eye discharge, and ear tilt as previously described [6]. The ability to induce a cough was assessed by entering the calf hutch and applying pressure to (while massaging) the bovine larynx. A calf with a cumulative score ≥ 5 was enrolled in the study as a BRDC case, and immediately thereafter, a calf adjacent to the BRDC case that possessed a cumulative score ≤ 3 was enrolled as a matched control. If no adjacent calf was suitable, the closest calf in the same row was enrolled as a matched control. Other observed clinical signs (though not included in the scoring system) were status for depression, dyspnea, tachypnea, and watery diarrhea, given the presence of fresh stool. Once enrolled, calves were assigned a case number and the hutch was labeled for subsequent monitoring. After enrollment, calves were observed and scored daily for clinical signs of BRDC. If a previously enrolled control calf converted to a BRDC case, its phenotypic classification was changed from control to a case and two new matched control and case calves were enrolled.

### Sample collection

Each day after completion of respiratory scoring, with identification of cases and matched controls, a veterinarian and facility personnel returned to those animals to collect samples. All case and control calves had nasopharyngeal and pharyngeal recess swabs collected for qPCR diagnostics, and a second pharyngeal recess swab was collected for aerobic bacteria and mycoplasma respiratory pathogen culturing. Samples collected from the nasopharyngeal region utilized a six-inch sterile unguarded polyester swab that was inserted five inches into a clean naris and rotated against the surface for 15 seconds. Thereafter, the swab was removed and cut ¼ inch above the polyester tip, and placed into 3 ml of viral transport media (minimum essential media, HCO_3_, HEPES, Gentamycin, Amphotericin B). Pharyngeal recess swabbing utilized a 27-inch sterile guarded swab with a polyester fiber tip (Kalajian Industries, Signal Hill CA 90755). The distance from the nose to the medial canthus of the eye was measured and marked as a gauge on the guarded swab. The swab was inserted through a nostril into the ventral meatus and then advanced toward the pharyngeal recess until reaching the gauge mark at the external naris. The swab was then pushed to open the guarding cap and advanced approximately two inches, rotated against the pharyngeal recess surface for 15 seconds, retracted back into the guarding sheath, and removed from the nose. The swab was cut two inches above the polyester tip, and placed into the same tube of viral transport media containing the nasopharyngeal swab. A second deep pharyngeal swab was placed into 1 ml of bacterial transport media (Brucella broth with 15% glycerol) for the detection of bacteria. All samples were stored on wet ice in the dark until completion of sampling and delivery to the lab. Scissors used to cut individual swabs were disinfected with a dilute Povidone iodine solution, and dried before each use. Bacterial samples were submitted daily for bacterial respiratory pathogen culture to either the California Animal Health and Food Safety lab system (CAHFS, Tulare CA Branch) for the CA samples, or to the Washington Animal Disease Diagnostic Laboratory at Washington State University (WADDL, Pullman, WA) for the NM samples. Viral qPCR samples from CA and NM were stored at -80°C until submission and processing at the CAHFS Davis Branch. Whole blood and serum were also collected for use in the GWAA studies.

### Analyses of animal data

The difference in mean age of groups of calves was tested using a *t*-test. Likewise, health scores between calf groups were investigated for differences using the Wilcoxon Rank-Sum test. To test whether the proportions of cases in groups of males and females were unequal, the standard test for two binomial proportions with continuity correction was used. All statistical tests were two-tailed tests with significance thresholds of *P* = 0.05 and were performed in R. A detailed summary of all tests is provided in the Additional file 1. The findings reported in Additional file 1 indicate that age and gender had an effect on susceptibility to BRDC, and therefore, were included in the GWAA models. Neither season nor month of sampling had an effect on BRDC susceptibility, and thus were not included in the GWAA models.

To date, conflicting results have emerged regarding the potential influence of gender on susceptibility to BRDC. Male beef calves have been reported to be more susceptible to BRDC than female calves, both at pre-weaning (14.4% compared to 9.5%) and post-weaning (17.2% compared to 12.5%) [17]. In the feedlot, Gallo and Berg [65] found that male calves were more likely to relapse (*P* = 0.0015) and be chronically affected (*P* = 0.03) with BRDC than females, and Cusack et al. [66] found that steers were more likely (*P* = 0.03) to die in the feedlot due to BRDC than were cows. In contrast, Ribble et al. [67] found that heifers were at a higher risk of death from BRDC than male calves, but in this study, the heifers were purchased in smaller groups than were their male counterparts, which may have impacted their risk for death. Finally, Sanderson et al. [68] reported no gender differences with respect to BRDC susceptibility, indicating a lack of unity within the established literature regarding the impact of gender on risk, and therefore, gender should be included within GWAA models for BRDC.

### Aerobic bacteria and Mycoplasma culture and qPCR

Culturing for aerobic respiratory pathogens allowed for the identification of the following important bacterial species: *Histophilus somni, Pasteurella multocida, Bibersteinia trehalosi, Mannheimia haemolytica*. The culture and qPCR of bacteria and mycoplasma for samples derived from the CA calves were conducted at the California Animal Health and Food Safety Laboratory System (University of California Veterinary Medicine Teaching and Research Center in Tulare, California). Similar samples obtained from the NM calves were processed using standardized procedures at WADDL (Pullman, WA). Additional aerobic culture methods are presented in the Additional file 1.

### Virology

Diagnostics for bovine corona virus, bovine respiratory syncytial virus, bovine viral diarrhea virus and bovine herpes virus type I were conducted from an aliquot of 3 ml of transport media (previously described) contained in 5 ml cryotubes that housed a mid-nasal and deep pharyngeal swab taken from each calf. Swabs were placed into the cryotubes immediately post collection and kept on ice until they reached the laboratory where they were frozen until analyzed. For the diagnostic analysis, samples were thawed and an aliquot of the transport media was removed for use in the qPCR diagnostics. Diagnostics were performed at the University of California Animal Health and Food Safety Laboratory System in Davis California.

### Analyses of diagnostic data

The estimated odds ratio for BRDC pathogens and case status (inclusive of the 95% confidence intervals for the estimated odds ratio) were calculated in R. A two-tailed Fisher’s exact test was used to determine if the odds ratios differed significantly from 1, with a significance threshold of *P* = 0.05.

### Bovine DNA isolation

Bovine DNA was isolated from 3 ml of whole blood collected in EDTA tubes using the Puregene DNA extraction kit, according to the manufacturer’s instructions (Gentra, Minneapolis, MN). DNA samples were quantified using NanoDrop (Wilmington, DE), spectrophotometry and purity was estimated using the 260/280 ratio. Samples with 260/280 ratios between 1.8 and 2.0 were diluted to 50 ng/μl, and 250 ng of each calf’s DNA was genotyped using the Illumina BovineHD Genotyping Beadchip at GeneSeek (Lincoln, NE). The Illumina (San Diego, CA) BovineHD Beadchip assay [69] contained 777,962 SNPs, with mean and median intermarker spacing of one SNP every 3.43 and 2.68 kb across the bovine genome, respectively.

### Heritability estimation

For the GBLUP analyses, custom developed software was used to generate the genomic relationship matrix [23] and to fit a generalized linear mixed model incorporating fixed effects and a random additive genetic merit effect for each individual. The software incorporates the inverse of the genomic relationship matrix and iterates on REML estimates of additive genetic and residual variance components until the heritability estimate has converged to a user-defined precision (*P* < 0.0005 here). At this point, GBLUP estimates of SNP effects were estimated as the regression of SNP effects on additive genetic merits as described by Taylor [70]. Pseudo-heritability [21] was similarly estimated using a genomic relationship matrix [23], with REML estimates of variance components, as previously described and implemented in Python [22].

### Genome-wide association analyses

Susceptibility to BRDC was treated as a binary trait based on the classification of the health scores obtained from the McGuirk [6] scoring system taken at the time of sample collection from the calves. The GWAA was conducted using four analytical approaches: 1) Efficient Mixed-Model Association eXpedited-genomic relationship matrix (EMMAX-GRM) [21, 22], 2) Full versus Reduced model (FvR) regression using principle component analysis-corrected input data within SNP & Variation Suite 7 (SVS; Golden Helix, Bozeman) [71], 3) GBLUP [70], and 4) EIGENSTRAT [72].

Using the binary BRDC case–control phenotype, the EMMAX-GRM analyses were conducted using an additive model and consisted of single marker tests of association [21], with corresponding estimates for the proportion of variance explained (PVE) by effects at single markers, and utilized a genomic relationship matrix [23], rather than an IBS kinship matrix [21, 22]. EMMAX-GRM analyses treated additive effects at X-linked loci in a different manner to those of autosomal loci, which has previously been suggested [70]. Specifically, within an additive model, hemizygous males may only possess 0 or 1 copy of an X-linked minor allele (excluding the pseudoautosomal region), whereas females may possess 0, 1, or 2 copies of the minor allele. A gender correction reflecting these ploidy differences was utilized to recode X-linked genotypes prior to EMMAX-GRM analyses. All EMMAX-GRM analyses were coded in Python [22, 73, 74] and were executed in the SVS environment (Golden Helix, version 7.7.6), thereby allowing for simultaneous data visualization and exploration while also providing a Python shell that allows for modification of scripts, as needed (i.e., REML versus ML variance component estimates, etc.). Briefly, the general mixed model can be specified as: *y* = *Xβ* + *Zu* + *ϵ*, where *y* is an *n* × 1 vector of the observed phenotypes, *X* is a *n* × *q* matrix of fixed effects, *β* is a *q* × 1 vector representing the levels of the fixed effects, and *Z* is a *n* × *t* matrix relating the instances of the random effect to the phenotypes. Herein, we assume that  and , such that , but in this study, *Z* is simply the identity matrix *I*, and *K* is the matrix of pairwise genetic relationships among all samples. To solve the mixed model equations using the generalized least squares solution, the variance components  must first be estimated (21–22). We used the REML-based EMMA approach to estimate the variance components as previously described [75], with stratification among all Holstein dairy calves accounted for and controlled using a GRM [23] computed from the filtered Illumina 778 K genotypes. Individual EMMAX-GRM analyses for NM and CA included both sex and age as covariates. Likewise, for our analysis of the combined cohort (NM + CA), both sex and age were again treated as covariates, but we also included a population of origin variable as a third and final covariate (Additional file 2: Figure S1). All relevant formulae are also available online (http://doc.goldenhelix.com/SVS/7.7.6/mixed_models.html; http://doc.goldenhelix.com/SVS/latest/mixed_models.html).

The FvR regression analysis utilized the binary BRDC case–control phenotype as the dependent variable within an additive model, with all genotypes recoded numerically based on the copy number of the minor allele. Recoding also included the same ploidy-based gender correction for X-linked loci described for the EMMAX-GRM procedure above. We corrected for population stratification using principal components analysis (PCA), with PCA-based correction of the input data using established methods [72, 76]. To identify a reasonable number of principal components for correction of the input data, we first estimated the Genomic Inflation Factor Lambda (λ) [77] across increasing numbers of principal components for each Holstein population, with 9 (NM), 53 (CA) and 91 (CA + NM) principal components yielding Inflation Factors (λ) of 1.06, 1.12, and 1.06, respectively. Thereafter, we implemented the FvR method, which consists of using two regression models commonly referred to as: A) the reduced model and B) the full model; with the significance of the genetic marker estimated after removing the effects of covariates by performing a full versus reduced model analysis. The reduced model included only the dependent variable (BRDC binary case–control status) and any potentially confounding covariates (i.e., the covariates sex, age, or population of origin variable in the combined NM + CA cohort). The full model included all of the variables plus the effect of each genetic marker individually. For the “full versus reduced” linear regression model (i.e., FvR), the regression sums of squares were calculated both for the reduced and for the full model. An F test was performed to find the significance of the full versus the reduced model as follows:


The *P*-value is calculated by: P-value = P(X > F*) where *X ~ (m-r,n-m)*, with *m* and *r* being the rank of the full and reduced model coefficient matrices, respectively, and *n* being the number of observations. All relevant formulae for PCA correction of input data, the FvR method, and recoding procedures have previously been described [71, 78]. For all FvR regression analyses, we used the following covariates: NM (sex, age), CA (sex, age), Combined cohort NM + CA (sex, age, population of origin variable) (Additional file 3: Figure S2). All PCA correction [72] and FvR regression analyses were performed in the SVS environment (Golden Helix, version 7.76), with relevant formulae available online (http://doc.goldenhelix.com/SVS/latest/formulas_theories.html#ftfullvreduced; http://doc.goldenhelix.com/SVS/latest/principal_component_analysis.html; http://doc.goldenhelix.com/SVS/latest/principal_component_analysis.html?highlight=pca%20corrected%20input%20data).

In the GBLUP analyses, we fit the model y_ijk_ = μ + Sex_i_ + βAge_j_ + a_k_ + e_ijk_ where y_ijk_ is a binary variable set to 1 for cases and 0 for controls of the i^th^ sex (i = male, female), j^th^ age (in days) and with additive genetic merit for susceptibility to BRDC of a_k_. The e_ijk_ are residuals assumed identically and independently distributed with null mean and for e = {e_ijk_} we assumed Var(e) =. For a = {a_k_} we assumed Var(a) =  where G is the genomic relationship matrix [23, 70]. Variance components  and  were estimated by restricted maximum likelihood and at convergence of the variance component estimates, SNP effects were estimated by GBLUP as the regression of SNP effects on additive genetic merits as described in Taylor [69]. The GBLUP analysis also included X-linked SNP using the approach described in [69]. Previous analyses using the software have been published including Decker et al. [79] and McClure et al. [80].

EIGENSTRAT [72] is a method for analysis of association between genotype and phenotype that corrects for stratification using a principal components approach. Principal component analysis is applied to genotype data to infer axes of genetic variation. Genotypes and phenotypes are subsequently adjusted using the top principal components. The adjusted genotype (*g*_*ij*__*adjusted*_) for each principal component is computed by:


where *g*_*ij*_ denotes genotype of individual *j* at SNP *i* and *a*_*j*_ denotes ancestry of individual *j* along a given principal component. The phenotypic adjustment is similarly computed for each principal component as:


Finally, the association between the adjusted genotypes and phenotypes is tested using the test statistic:


where *r*_*i*_ is the correlation between the adjusted genotype at SNP *i* (*g*_*ij, adjusted*_) and the adjusted phenotype (*p*_*j, adjusted*_), *N* is the number of individuals, and *K* is the number of principal components used for adjustment. This test statistic is χ^2^ distributed with one degree of freedom.

In order to correct for the stratification in the genotypic data, the first (top) 100, 5, and 80 principal components were used in the EIGENSTRAT analysis of California, New Mexico, and the combined data sets, respectively. The use of these principal components also corrected for the relatedness (sire half-sib families) that was found among the calves (Additional file 4: Figure S3, Additional file 5: Figure S4). As a result, the Genomic Inflation Factor Lambda (λ) values declined to 1.08 in CA, 1.09 in NM, and 1.09 in the CA-NM combined data. Sex and age were fit in the model for CA, age was fit in the model for NM and sex, age was fit in the model for the combined population (Additional file 6: Figure S5).

Significance for all association tests (i.e., EMMAX-GRM, FvR, EIGENSTRAT) was based on the recommendation of the Wellcome Trust Case Control Consortium [81] where unadjusted *P*-values less than 5 × 10^-7^ were considered to provide strong evidence of association, and unadjusted *P*-values between 5 × 10^-5^ and 5 × 10^-7^ were considered to provide moderate evidence for association. Individual analyses were conducted for the CA and NM populations, with final analyses that included the combined cohort (NM + CA). For comparisons of the top SNPs and loci identified across the four analytical approaches, the top ranked 2,000 SNPs from each of the four analytical approaches were identified and compared. SNPs were ranked either by *P* value (EIGENSTRAT, EMMAX-GRM and FvR) or by PVE (GBLUP) from 1 (most significant SNP) to 2,000. For comparisons of SNPs that were associated with BRDC susceptibility across all statistical methodologies (EIGENSTRAT, EMMAX-GRM, FvR and GBLUP) as shown in Tables 3, 4 and 5, the ranking of the SNP in each method was summed to arrive at a composite ranking. SNPs with a composite ranking that was less than 1000 across all methods are provided in Tables 3, 4 and 5.

QTL were defined by the number of top ranked SNPs that were associated with BRDC susceptibility for a genomic region within each statistical method. For example, if a single SNP within a region was among the top ranked SNPs, the locus would be defined by the single SNP. However, if several highly ranked SNPs were in close proximity to one another, the QTL would be defined by the chromosomal location of the outermost SNPs in that region. An example of this would be the QTL on BTA4 (48–49 Mb) in the CA + NM population where the QTL is defined by 7 SNPs within the region 48,052,484 to 48,149,487 bp.

## Authors’ information

The Bovine Respiratory Disease Complex Coordinated Agricultural Project Research Team - Team members include: David Anderson, Noah Cohen, Allan Dabney, Scott Dindot, Mark Enns, Laurel Gershwin, Joseph S Neibergs, Tim Ross, Loren Skow, Milton Thomas, Cassandra Tucker, Curt Van Tassell, and Aldroaldo Zanella.

## Electronic supplementary material

Additional file 1: **Supplementary Information.** **Table S1.** Distribution of pre-weaned Holstein calf health scores (McGuirk 2008). **Table S2.** Top 30 ranked bovine SNPs by the EMMAX-GRM analyses of the California, New Mexico and combined populations. **Table S3.** Top 30 ranked bovine SNPs for GBLUP analyses of California, New Mexico and combined populations. **Table S4.** Top 30 ranked bovine SNPs resulting from the FvR analyses of California, New Mexico and the combined populations. **Table S5.** Top 30 ranked bovine SNP for EIGENSTRAT analyses of California, New Mexico and combined populations. (PDF 817 KB)

Additional file 2: Figure S1: EMMAX-GRM P-P plots with age and sex in the model for: A. California, and B. NM. In panel C., sex, age and population of origin were included in the combined CA-NM population. For each of the plots in panels A-C, the observed –log10 P-value (on the Y axis) is plotted against the expected –log10 P-value (on the X axis). (PDF 126 KB)

Additional file 3: Figure S2: FvR P-P plots with sex and age in the model for: A. California with correction for 53 principle components, and B. NM calves with correction of 9 principle components. In panel C. sex, age and population of origin were included in the combined CA-NM population which included correction of 91 principle components. For each of the plots in panels A-C, the observed –log10 P-value (on the Y axis) is plotted against the expected –log10 P-value (on the X axis). (PDF 128 KB)

Additional file 4: Figure S3: The first four EIGENSTRAT principal component analyses plots showing the distribution of presumed half-siblings (based on the genomic relationship matrix) of ten sires with the most offspring within the California calf study population. Each sire’s offspring are coded with a different color and tend to cluster together within the study population. In panel A., the first principal component (PC1) (plotted on the X axis) is compared against the second principal component (PC2)( on the Y axis). In panel B., PC1 is again plotted on the X axis but is now compared against the third principal component (PC3) on the Y axis. Principal component 2 (PC2) (on the X axis) is plotted against the third principal component (PC3)( on the Y axis) in panel C. In Panel D., PC1 (on the X axis) was plotted against principal component 4 (PC4) (on the Y axis). In panel E., PC2 (on the X axis) was compared to PC4 (on the Y axis). Finally, in panel F., PC1 was plotted (on the X axis) and compared to PC4 (on the Y axis). (PDF 816 KB)

Additional file 5: Figure S4: The first four EIGENSTRAT principal component analyses plots showing the distribution of presumed half-siblings (based on the genomic relationship matrix) of the ten sires with the most offspring within the New Mexico calf population. Each sire’s offspring are coded with a different color and tend to cluster together within the study population. In panel A., the first principal component (PC1) (plotted on the X axis) is compared against the second principal component (PC2)( on the Y axis). In panel B., PC1 is again plotted on the X axis but is now compared against the third principal component (PC3) on the Y axis. Principal component 2 (PC2) (on the X axis) is plotted against the third principal component (PC3)( on the Y axis) in panel C. In Panel D., PC1 (on the X axis) was plotted against principal component 4 (PC4) (on the Y axis). In panel E., PC2 (on the X axis) was compared to PC4 (on the Y axis). Finally, in panel F., PC1 was plotted (on the X axis) and compared to PC4 (on the Y axis). (PDF 360 KB)

Additional file 6: Figure S5: EIGENSTRAT Q-Q plots: A. CA with correction of 100 principle components and age and sex included in the model, B. NM calves with correction of 5 principle components with only age included in the model, and C. the combined CA-NM population with correction of 80 principle components and sex and age included in the model. For each of the plots in panels A-C, the observed –log_10_ P-value (on the Y axis) is plotted against the expected –log_10_ P-value (on the X axis). (PDF 169 KB)
