# Supplementary material for: Susceptibility loci revealed for bovine respiratory disease complex in pre-weaned holstein calves
Source: BMC Genomics. 2014 Dec 22;15(1):1164. doi: 10.1186/1471-2164-15-1164 (PMC4445561; doi:10.1186/1471-2164-15-1164)
Supplement: Supplementary file 1 — Additional file 1: Supplementary Information. Table S1. Distribution of pre-weaned Holstein calf health scores (McGuirk 2008). Table S2. Top 30 ranked bovine SNPs by the EMMAX-GRM analyses of the California, New Mexico and combined populations. Table S3. Top 30 ranked bovine SNPs for GBLUP analyses of California, New Mexico and combined populations. Table S4. Top 30 ranked bovine SNPs resulting from the FvR analyses of California, New Mexico and the combined populations. Table S5. Top 30 ranked bovine SNP for EIGENSTRAT analyses of California, New Mexico and combined populations. (PDF 817 KB) [file 12864_2014_7079_MOESM1_ESM.pdf]

Supplementary Information

Study Animals

The California (CA) population consisted of 2,021 Holstein calves. Seven calves were removed due to discrepancies in phenotypes leaving 2,014 calves enrolled as cases (n = 1,003) and controls (n = 1,011), with 1,257 males and 757 females. The calf facility housed 80,000 animals, and all Holstein calves were obtained from dairies in the Central Valley of CA, either for custom heifer raising, or to raise as steers for sale to feedlots. All CA calves were raised in individual hutches that allowed casual contact between adjacent calves, with production rows consisting of 480 individual hutches. New arrivals ranged in age (i.e., from 24 to 48 hours old), and were placed in sanitized hutches. Ranch personnel assessed all new arrivals for failure of passive transfer by evaluating serum total proteins with a refractometer. Husbandry of the calves at the facility included feeding of milk replacer twice daily, with grain and water provided *ad libitum*. Newly arrived calves were vaccinated intranasally against infectious bovine rhinotracheitis (IBR) as well as bovine parainfluenza virus 3 (PI3), and at 8 days of age, were also given a 5-way modified-live parenteral vaccine consisting of IBR, bovine viral diarrhea virus 1 and 2 (BVDV), PI3, and bovine respiratory syncytial virus (BRSV). *Moraxella bovis* bacterin was given at 65 days of age. Weaning occurred between 60 to 75 days of age before transition to group pens, based on capacity and new animal influx at the ranch.

The overall range and frequency distribution of the McGuirk (2008) [6] health scores observed for the CA calves are provided in **Supplementary Table 1**. The mean health score for controls

was 1.1, with a standard deviation of 0.81 (± 0.81), and was 6.6 ± 1.36 for BRDC cases. Moreover, almost 94% of the control calves had a health score of 2 or less, whereas 77% of the cases had scores between 5 and 8. Of the control animals that eventually converted to a case, the mean health score as controls was 1.3 ± 1.01, which was similar to the mean of all controls (P = 0.35). The mean health scores of the male cases was 6.52 ± 1.37, which was similar to the health scores for female cases (6.6 ± 1.34, P = 0.27). In contrast, the mean health score of the male controls was lower (1.06 ± 0.82,) than for the female controls (1.2 ± 0.8, P = 0.0035).

The mean age of the CA Holstein BRDC cases at diagnosis was 46.4 ± 8.5 days, which was very similar to that of the controls (47.9 ± 8.5 days). The mean age of the cases and controls at sampling differed (P = 0.0001) for the CA calves. The mean age of the male cases was 45.9 ± 8.0 days, which differed (P = 0.03) from the mean age of the female cases (47.1 ± 9.1). Among controls, the mean age of males (47.9 ± 8.4 days) did not differ (P = 0.96) from females (47.8 ± 8.7 days). Likewise, there was no difference (P = 0.10) in the proportion of cases among males (48.37%) and females (52.18%).

All of the NM calves were recorded as being anatomically female and were housed in one of three calf raising facilities. Samples were collected at three calf raising programs that received calves from multiple dairy farms. The first calf raising facility received calves from 7,000 of their own dairy cows raised at three nearby locations. These calves were housed in California-style hutches facing to the south on a gradual slope to facilitate drainage. In the winter the hutch tops were lowered and the bottoms boarded up to help protect the calves from the wind. Calves were watered and assessed three times per day and fed twice a day with heated milk

replacer. Calves were given colostrum at birth and a gallon per day for the next three days. Calves were vaccinated intranasally against IBR, PI3 and BRSV. *Salmonella typhimurium* bacterin was given at 5 and 12 days of age. Vaccination against *Moraxella bovis* was given at day 21 with clostridium Chauvoei-Septicum-Novyi-Sordelli-Perfringens Types C &D bacterin administered at day 28. Calves were weaned at 60 days of age.

The second NM calf raising facility was similar in that calves originated from 13,000 cows from 3 dairies. These calves received a gallon of colostrum via esophageal tube and were intranasally vaccinated against IBR as well as PI3, with vitamin B12 and Penicillin administration upon arrival. Calves were housed in bungalow-style hutches made of wood that faced south on a slope and had a small wire panel yard attached to the hutch. Calves were watered twice a day and assessed once a day. Calves were fed a mixture of hospital milk and milk replacer. At 30 days of age calves were vaccinated with a modified live culture of *Pasteurella haemolytica* and *Pasteurella multocida*, and at 45 and 70 days of age calves received a modified live vaccine for IBR, BVD types I and II, PI3 and BRSV. Calves we4re weaned at 70 days of age.

The third NM calf raising facility received calves from a single 3000 cow dairy. Calves were housed in plastic hutches, each with a wire yard attached to it. Hutches were not set on a slope and were oriented east during the summer months and south during the winter months. Calves were given 1 gallon of colostrum within the first hour of birth, and 2 quarts of colostrum were given the following morning and afternoon. Calves were fed and watered twice a day. Milk, consisting of pasteurized fresh milk and milk replacer, was heated and fed to the calves twice a day. Calf health was assessed twice a day. The vaccination protocol for this facility included an

intranasal modified live vaccine for IBR and PI3 the week the calves were received, a modified live vaccine for IBR, BVD types I & II, BRSV, PI3, *Mannheimia haemolytica* and *Pasteurella multocida* during weeks 2 and 6, *Moraxella bovis* bacterin vaccine at week 4, and clostridium Chauvoei-Septicum-Novyi-Sordelli-Perfringens Types C &D bacterin was administered at week 7. Calves were weaned at 80 days of age.

The mean health scores of the control heifers in NM were 1.7 ± 0.9, and 8.3 ± 1.5 for the cases. The mean health score for controls (that ultimately converted to cases) at the tim87)e of selection was 1.6 ± 1.2, as compared to mean scores of 8.1 ± 1.5 after conversion and reclassification to case status. Mean health score for the CA and NM controls differed (P = 0.001), as did the mean health score for the cases (P < 0.0001), which was due to higher scores in NM (**Supplementary Table 1**). The proportion of control calves that converted to cases was not different (P = 0.61) between CA and NM. The mean age for calves diagnosed as a BRDC case was 53.5 ± 13.0 days, which did not differ (P = 0.7) from the mean age of the matched controls when sampled (53.1 ± 11.6 days). The mean age at which the calves were sampled was different between the California and New Mexico populations for both cases and controls (P < 0.0001).

The combined California and New Mexico population consisted of 2,763 Holstein calves; 1,505 females and 1,258 males, which included 1,384 controls and 1,379 cases. The mean health score for controls and cases was 1.3 ± 0.9 and 7.1 ± 1.6, respectively. The mean case and mean control health scores for the combined population differed from the California (P < 0.0001) and the New Mexico (P < 0.0001) populations. The mean age of the combined cases and controls differed (P = 0.01) between the ages of the cases and controls in the combined population. The

mean age for the combined cases was 48.3 ± 10.4 days and 49.3 ± 9.7 for the combined controls.

Diagnostics

The two most common organisms detected in the cases were *Mycoplasma* spp. (64.6% in CA, 57.4% in NM), and *Pasturella multocida* (36.3% in CA, 61%in NM). These two organisms were also the most frequently detected in the controls. *Mycoplasma* spp. was detected in 57.1% of controls in CA and 48.7% of controls in NM, whereas *Pasturella multocida* was detected in 23.6% and 54.8% of controls in CA and NM, respectively. *Mycoplasma spp.* were the most common organism detected in the combined CA and NM population for both cases (62.6%) and controls (54.8%), followed by *Pasturella multocida*, with 43% of cases and 32% of controls identified as being diagnostically positive for this organism.

The proportions of the remaining pathogens detected in each population were dissimilar among cases and controls. All detected pathogens were found to be present as commensal organisms (at some level) among the controls, with the exception of bovine viral diarrhea virus and bovine herpes viruses 1 & 2, which were either not detected in the cases (bovine herpes virus) or were detected in only very few individuals (bovine viral diarrhea virus). The odds ratio, computed as the odds of BRDC given the presence of a pathogen in the combined CA and NM population, ranged from 1.4 for *Mycoplasma* spp., to 4.9 for *Histophilus somni*. The relatively small number of calves for which *H. somni* was detected may have resulted in an inflation of the odds ratio. The mean odds ratio for all seven detected pathogens was 2.5.

Aerobic Bacteria and Mycoplasma Culture and qPCR

For the CA samples, aerobic bacterial cultures were performed by plating the transport media (Brucella broth) from the mid nasal and deep pharyngeal swabs onto sheep blood agar and chocolate agar for 48 hours at 37°C. Initial typing of organisms was based on colony morphology, and subsequently confirmed with biochemical testing. *Mycoplasma* spp. were cultured in an enrichment broth for 48 hours, plated on modified Hayflick media, and grown in a CO_2 incubator at 37°C for 7 days. Mycoplasma species were identified based on colony morphology and confirmed with digitonin and Diene's Stain. Quantitative PCR for respiratory pathogens was set up to identify bovine herpes virus-1 (BHV-1) [82], BVDV [83], BRSV [84], and bovine coronavirus (unpublished data, Wisconsin Veterinary Diagnostic Laboratory, University of Wisconsin, Madison, WI 53706).

For the NM samples, deep pharyngeal swabs were placed in a 10% glycerol Brucella broth transport media and kept on ice and transported to the Bacteriology Section at Washington Animal Disease Diagnostic Laboratory. Swabs were used to inoculate the glycerol media in Columbia Blood Agar (CBA) plates and Columbia Selective Agar (CSA, selective medium used for the isolation of *Pasteurella*, *Mannheimia*, *Bibersteinia*, and *Histophilus* spp). Mycoplasma broth was also inoculated by pouring in the glycerol media. CBA, CSA and mycoplasma broth were incubated at 35 ˚C in CO_2. CBA and CSA were observed for two days, growing colonies that phenotypically indicated the presence of members of the *Pasteurellaceae* family, *Streptococcus* spp with alpha haemolysis, *Trueperella pyogenes* and others. Non-

fermenter Gram-negative rods were isolating in CBA and MacConkey agar and incubated for 24 - 48 hours at 35˚C in CO_2. Genus and specie identification was based on biochemical tests.

After 3 – 5 days of incubation the mycoplasma broth was sub-cultured in mycoplasma plates with a Dacron swab, and a digitonin disk was placed into the agar surface. Mycoplasma plates were incubated for 48 – 72 hours at 35˚C in CO_2. After incubation, the inhibition zone around the digitonin disk was measured and Diene's stain was performed. If the inhibition zone was ≥ 15mm and the Diane's stain showed the classical mycoplasma colony morphology, the microorganism was identified as *Mycoplasma* spp. The mycoplasma broth was sent to PCR for specie identification.

Quality Assurance

For the EIGENSTRAT analyses, quality control filtering for the 2014 California calves for the EIGENSTRAT [72] analysis resulted in the removal of animals with a genotyping call rate < 95% (n=46). In addition, 5 animals were identified as Klinefelter XXY calves and 3 calves possessed a phenotypic gender that differed from that predicted by their genotypes, resulting in their removal from the study. The mean genotype call rate for the remaining 1,958 calves (1,225 males and 733 females from the California population) was 99.3%. Of the 739 New Mexico calves, 2 calves were identified as putative Klinefelter calves and removed, and 2 were removed due to gender differences between their reported phenotypes and genotypes. Forty-seven animals were removed for possessing a genotype call rate less than 95% leaving 344 cases and 348 controls with a mean genotype call rate of 99.4%. Quality control filtering of SNPs for the EIGENSTRAT analysis was performed individually for each population, and removed any SNPs

with minor allele frequency (MAF) < 0.01, a call rate < 0.9, or that deviated from Hardy-Weinberg equilibrium with a $P < 1 \times 10^{-100}$. After filtering, a total of 619,503 SNPs for the CA calves and 628,925 SNPs for the NM calves remained for association analysis.

Prior to performing EMMAX [21, 22] using a genomic relationship matrix (GRM) [23] and FvR [71] regression analyses, all dairy calf genotypes (CA + NM) and diagnostic data were combined into a single file for quality control analyses and filtering. Calves possessing an overall genotype call rate < 0.90 were removed. Thereafter, we filtered SNPs with MAF < 0.001, a call rate < 0.85, and/or those that deviated from Hardy-Weinberg Equilibrium (Fisher's exact HWE $P < 1 \times 10^{-100}$). Calves with gender, birthdate, and/or other disparities were also removed. The potential for male gender disparities were initially predicted by X chromosome heterozygosity > 0.02. Calves exhibiting overt evidence of Klinefelter's syndrome or technical problems, as evidenced by extensive X chromosome heterozygosity and high Y chromosome SNP call rates (> 0.50), were subsequently removed. However, two NM calves originally recorded as males following field observations produced evidence for X chromosome heterozygosity > 0.02. While both calves produced Y chromosome genotypes, they possessed less X chromosome heterozygosity than 99.9% (n = 704) of all phenotypically unambiguous NM females (n = 705), suggesting that this result may be due to missassemblies of the X chromosome, expansions/duplications, or other unknown anomolies. We conducted EMMAX-GRM and FvR analyses treating these two calves alternately as both male and female for comparison. At the conclusion of our quality control analyses, 2,682 Holstein calves (n = 1,975 CA; n = 707 NM) and 651,637 SNPs remained for BRDC case-control GWAA using EMMAX and FvR regression. The

final ratio of cases to controls was nearly 1:1 in both NM (n = 352 cases, n = 355 controls) and CA (n = 981 cases, n = 994 controls), which collectively included 1,246 males and 1,436 females.

To assess potential population stratification that may lead to spurious GWAS results, principal component analyses (PCA) (i.e., see **Supplementary Figures 1** and **2**), quantile-quantile (Q-Q) (**Supplementary Figure 3**) and probability-probability (P-P) (**Supplementary Figures 4** and **5**) plots were used. In addition, genomic inflation factors were calculated to evaluate population stratification in each of the individual and the combined populations. The PCA for EIGENSTRAT was conducted using a reduced number of SNPs (185,481 SNPs for California, 201,552 SNPs for New Mexico and 187,609 SNPs for the combined data sets) which were obtained using the LD pruning procedure implemented in PLINK [85]. This reduction in SNPs decreased the level of LD between SNPs and reduced the squared correlation between SNPs to $r^2 < 0.75$. The PCA and genomic inflation factors were computed using EIGENSOFT [72]. The Q-Q plots were created in R where the expected *P*-value (for each SNP) was compared to the observed *P*-value on a -log10 scale.

For the GBLUP analyses [70], quality control filtering of SNPs was also performed jointly for both the CA and NM populations. All SNPs with MAF < 0.001, with a call rate < 0.85, or that deviated from Hardy-Weinberg equilibrium with a statistic of $\chi_1^2 > 100$ ($P < 1.52 \times 10^{-23}$) were removed. These edits left a total of 654,044 SNPs for both the California and New Mexico calves. Animals were filtered if they possessed a genotype call rate < 0.90, had an mean autosomal heterozygosity > 0.40, or were detected to be putative Kleinfelter calves via having a non-paX (pseudo-autosomomal) chromosome heterozygosity > 0.03 and a Y chromosome SNP

call rate > 0.50. These filters left a total of 2,703 animals of which 1,239 were male and 1,464 were female. These filters resulted in a genotype dataset in which 0.55% of genotypes were missing values which were imputed using Beagle v3.3.2 [86].

Month/Seasonal Effects

In addition to evaluating the effects of age, gender, and the proportion of males and females in the case-control groups, we also evaluated season and month to determine if either had an effect on the loci identified as being associated with BRDC susceptibility. As it is well known that the prevalence of BRDC is affected by season, the study was purposely designed to eliminate any seasonal effects by equally sampling cases and controls within each month/season. This eliminates the potential confounding effect of differences in prevalence. However, the loci identified as being associated with BRDC susceptibility may be altered if the pathogens that elicit BRDC differ by season or month. This was tested for with EIGENSTRAT where month or season was coded as a categorical covariate and then the top 2000 SNP markers were compared to the analyses which did not include month or season. When comparing the overlap of the top 2000 SNP in the EIGENSTRAT analysis that didn't include season and the analyses that did, the concordance of the SNPs was 95.7% for CA, 90.5% for NM and 97% for the combined population. The same comparison for month the animal was sampled demonstrated that 89.6% of the CA, 90.6% of the NM and 94.1% of the combined population SNPs were shared between the analyses.

The potential effect of season and month on the GWAA results was also directly evaluated within the EMMAX-GRM and FvR approaches. The inclusion of month and season within an additive EMMAX-GRM model produced results that were highly concordant with a model that included only sex and age. Specifically, 96%-99% of the top 2000 SNPs were shared when season or month was either included or excluded in the EMMAX-GRM analyses for the CA, NM and combined populations. Moreover, all of the same top 30 SNPs (**Supplementary Table 2**) were identified as being associated with BRDC susceptibility regardless of whether season or month were included or excluded in the EMMAX-GRM model for all study populations. Inclusion of month or season also did not impact the pseudo-heritability estimates for CA, NM, or the combined cohort. Similarly, inclusion of season or month also had little impact on the FvR results, with the same top 30 SNPs identified as being associated with BRDC susceptibility **Supplementary Table 4**. When the top 2000 SNPs identified by FvR were compared before and after inclusion of season, 96.9% of the SNPs were shared in CA, 98.9% were shared with NM, and 97.5% were shared in the combined population. When the top 2000 SNPs from models that included and excluded month were compared, 98.3% of the SNPs were shared in CA, 95.9% were shared in NM, and 99.6% were shared in the combined population.

There was also no effect ($P > 0.05$) when month of sampling was included in the GBLUP analyses. However, when season was included in the GBLUP model, a nominally significant effect of season for CA was detected ($P = 0.02$), with no similar effect observed for NM or the combined population ($P > 0.05$). When further evaluated, the effect of season for CA was determined to be due to the difference in the mean age of animals across the seasons. In the summer in CA, the mean age of calves was 48.2 days, 48.4 days in the fall, and 40.6 days in the

winter. By including season in the GBLUP model, some of the age differences were removed, resulting in a significant result even though the effect was due to age. Therefore, the correct analytical model was to include age (and sex) in the additive model, but not an effect for season of sampling (**Supplementary Table 3**).

Supplementary Table 1. Distribution of pre-weaned Holstein calf health scores (McGuirk 2008).

Health Score*	# of Animals Receiving Score*			Binary Phenotype
	**California**[+] n = 1,941	**New Mexico^** n = 748	**Combined** n = 2,689	
0	216	34	250	Control
1	530	122	652	Control
2	210	160	370	Control
3	49	51	100	Control
4	6	5	11	Control
5	244	13	257	Case
6	264	27	291	Case
7	206	74	280	Case
8	126	86	212	Case
9	67	100	167	Case
10	18	45	63	Case
11	3	26	29	Case
12	2	5	7	Case

*0 represents no clinical symptoms of BRDC.
[+]Animals from California that converted from a control to a case (n = 73) are not included. ,
^Jersey calves (n = 18) were removed.

Supplementary Table 2. Top 30 ranked bovine SNPs by the EMMAX-GRM analyses of the California, New Mexico and combined populations.

Chromosome	Location (Mb)	*P* value for most significant SNP	Rank for 30 most significant SNPs*
**California**			
BTA15	30-31	$2.95x10^{-6}$	1
BTA26	49-50	$1.20x10^{-5}$	2
BTA23	3-4	$1.62x10^{-5}$	3-6,15
BTA18	0.8-0.9	$2.67x10^{-5}$	7-13, 17-23,25-28, 30
BTA14	63-64	$3.55x10^{-5}$	14
BTA14	10-11	$3.80x10^{-5}$	16
BTA27	15-16	$4.07x10^{-5}$	24
BTA4	4-5	$4.68x10^{-5}$	29
**New Mexico**			
BTA5	23-24	$1.22x10^{-5}$	1, 21, 22
BTA16	70-71	$1.39x10^{-5}$	2, 6, 8, 10-12, 14, 24, 28-30
BTA13	67-68	$2.27x10^{-5}$	3
BTA2	2-3	$2.91x10^{-5}$	4, 7, 15, 24, 28, 30
BTA14	7-8	$2.97x10^{-5}$	5
BTA18	63-64	$3.88x10^{-5}$	13
BTA6	85-86	$5.03x10^{-5}$	19
BTA24	9-10	$5.16x10^{-5}$	20
BTA13	53-54	$5.51x10^{-5}$	23
BTA4	64-65	$6.04x10^{-5}$	25-27
**California and New Mexico Combined**			
BTA15	30-31	$1.95x10^{-5}$	1
BTA11	80-81	$2.45x10^{-5}$	2
BTA8	73-74	$3.35x10^{-5}$	3, 10-12, 15-19
BTA4	47-48	$3.88x10^{-5}$	4-9, 13, 14, 20-24, 27, 28
BTA20	0-1	$5.29x10^{-5}$	25, 30
BTA17	16-17	$5.37x10^{-5}$	26
BTA26	12-13	$5.67x10^{-5}$	29

*Indicates the SNP with the ranking closest to 1. SNPs that are the highest ranked by an individual method, are not necessarily the SNPs that are ranked highest across all analyses as shown in **Tables 3-5.**

Supplementary Table 3. Top 30 ranked bovine SNPs for GBLUP analyses of California, New Mexico and combined populations.

Chromosome	Location (Mb)	Proportion of variance explained^	Rank of 30 most significant SNPs*
**California**			
BTA15	30-31	0.13	1, 8, 25-28
BTA23	3-4	0.13	2-6
BTA14	63-64	0.12	7, 17, 29
BTA3	119-120	0.11	9, 18
BTA29	35-36	0.11	10-16
BTA4	47-48	0.11	19
BTA6	42-43	0.11	20-22
BTA18	46-47	0.11	23
BTA2	45-46	0.10	29
BTA22	50-51	0.10	30
**New Mexico**			
BTAX	61-62	0.08	1
BTAX	142-143	0.07	2
BTA16	70-71	0.07	3-5, 8-11
BTA13	67-68	0.07	6, 14
BTAX	27-28	0.07	7, 26
BTA2	6-7	0.06	12
BTAX	21-22	0.06	13, 15
BTAX	26-27	0.06	16
BTA13	56-57	0.06	19
BTA13	53-54	0.06	20, 21, 24
BTA24	22-23	0.06	22
BTA28	36-37	0.06	23
BTA6	85-86	0.06	234
BTAX	55-56	0.06	27, 29
BTA16	64-65	0.06	28
BTA12	77-78	0.06	30
**California and New Mexico Combined**			
BTA15	30-31	0.11	1
BTA15	31-32	0.10	2
BTA17	16-17	0.10	3
BTA20[#]	0-1	0.10	4, 12, 15, 20
BTA20[#]	0-1	0.10	5 - 10, 13, 14, 16
BTA12	58-59	0.10	11, 21
BTA17	17-18	0.10	17,18
BTA4	47-48	0.10	19
BTAX	16-17	0.10	22
BTA29	35-36	0.10	28, 29
BTA17	15-16	0.10	30

^Proportion of additive genetic variance explained by a window of 7 adjacent SNPs

*Indicates the SNP with the ranking closest to 1. SNPs that are the highest ranked by an individual method, are not necessarily the SNPs that are ranked highest across all analyses as shown in **Tables 3-5.**

[#]Loci are separated by 200 kb.

Supplementary Table 4. Top 30 ranked bovine SNPs resulting from the FvR analyses of California, New Mexico and the combined populations.

Chromosome	Location (Mb)	*P* value for most significant SNP	Rank of 30 most significant SNPs*
**California**			
BTA15	30-31	3.74×10^{-7}	1, 23
BTA18	0-1	3.55×10^{-6}	2, 6, 8, 11-14, 16-18, 20-21, 25-28, 30
BTA14	11-12	5.23×10^{-6}	3
BTA14	10-11	5.99×10^{-6}	5
BTA3	119-120	6.6×10^{-6}	7
BTA26	49-50	7.8×10^{-6}	9
BTA14	62-63	7.84×10^{-6}	10
BTA14	63-64	1.09×10^{-5}	15
BTA15	14-15	1.31×10^{-5}	19
BTA3	15-16	1.37×10^{-5}	23
BTA3	88-89	1.4×10^{-5}	24
BTA14	82-83	1.56×10^{-5}	29
**New Mexico**			
BTA16	70-71	2.8×10^{-6}	1-3, 10-13, 17
BTA5	23-24	6.04×10^{-6}	4, 8, 9,
BTA6	87-88	6.56×10^{-6}	5-7
BTA8	63-64	1.64×10^{-5}	14, 15, 21-29
BTA1	3-4	2×10^{-5}	16, 20
BTA1	34-35	2.07×10^{-5}	19
BTA14	7-8	2.28×10^{-5}	30
**California and New Mexico Combined**			
BTA8	73-74	5.17×10^{-6}	1, 5-7, 10-14
BTA15	30-31	6.42×10^{-6}	2
BTA12	87-88	7.29×10^{-6}	3
BTA7	11-12	1.01×10^{-5}	4
BTA20	0-1	1.32×10^{-5}	9, 30
BTA18	46-47	1.7×10^{-5}	16
BTA3	119-120	1.8×10^{-5}	17, 21, 28, 29
BTA11	80-81	1.85×10^{-5}	18
BTA27	2-3	1.95×10^{-5}	19
BTA19	9-10	1.97×10^{-5}	20
BTA17	14-15	2.29×10^{-5}	26
BTA7	10-11	2.13×10^{-5}	22, 24, 25, 27

*Indicates the SNP with the ranking closest to 1. SNPs that are the highest ranked by an individual method, are not necessarily the SNPs that are ranked highest across all analyses as shown in **Tables 3-5.**

Supplementary Table 5. Top 30 ranked bovine SNP for EIGENSTRAT analyses of California, New Mexico and combined populations.

Chromosome	Location Mb	P value for most significant SNP	Rank of 30 most significant SNPs*
**California**			
BTA14	10-11	$1.47x10^{-6}$	1, 4, 7, 14, 15
BTA18	0-1	$2.24x10^{-6}$	2, 8-13, 16-22, 24-25, 29
BTA15	14-15	$4.28x10^{-6}$	3
BTA3	119-120	$6.24x10^{-6}$	5
BTA14	63-64	$6.47x10^{-6}$	6, 27,28
BTA26	49-50	$1.85x10^{-5}$	23
BTA15	30-31	$2.14x10^{-5}$	30
**New Mexico**			
BTA2	71-72	$6.35x10^{-6}$	1
BTA4	64-65	$6.98x10^{-6}$	2-4, 18
BTA8	72-73	$8.35x10^{-6}$	5
BTA14	7-8	$1.08\ x10^{-5}$	6, 7, 27, 28, 30
BTA19	32-33	$1.14x10^{-5}$	8
BTA16	70-71	$1.42x10^{-5}$	9, 23-26
BTA8	73-74	$1.65x10^{-5}$	10-13, 16, 17, 19-22
BTA12	77-78	$1.82x10^{-5}$	14
BTA29	46	$1.84x10^{-5}$	15
BTA13	71-72	$2.66x10^{-5}$	29
**California and New Mexico Combined**			
BTA19	9-10	$5.82x10^{-6}$	1
BTA4	48-49	$7.09x10^{-6}$	2-8
BTA26	12-13	$1.34x10^{-5}$	9
BTA11	80-81	$1.39x10^{-5}$	10
BTA15	30-31	$1.75x10^{-5}$	11
BTA14	62-63	$1.79x10^{-5}$	12
BTA8	73-74	$2.59x10^{-6}$	16-22
BTA4	6-7	$3.48x10^{-5}$	23
BTA15	82-83	$3.67x10^{-5}$	24
BTA15	31-32	$4.04x10^{-5}$	25
BTA18	55-56	$4.09x10^{-5}$	26, 29
BTA15	66-67	$4.2x10^{-5}$	27
BTA14	40-41	$4.48x10^{-5}$	28
BTA26	3-4	$5x10^{-5}$	30

*Indicates the SNP with the ranking closest to 1. SNPs that are the highest ranked by an individual method, are not necessarily the SNPs that are ranked highest across all analyses as shown in **Tables 3-5.**

Supplementary Figure 1 The first four EIGENSTRAT principal component analyses plots showing the distribution of presumed half-siblings (based on the genomic relationship matrix) of ten sires with the most offspring within the California calf study population. Each sire's offspring are coded with a different color and tend to cluster together within the study population. In panel A., the first principal component (PC1) (plotted on the X axis) is compared against the second principal component (PC2)(on the Y axis). In panel B., PC1 is again plotted on the X axis but is now compared against the third principal component (PC3) on the Y axis. Principal component 2 (PC2) (on the X axis) is plotted against the third principal component (PC3)(on the Y axis) in panel C. In Panel D., PC1 (on the X axis) was plotted against principal component 4 (PC4) (on the Y axis). In panel E., PC2 (on the X axis) was compared to PC4 (on the Y axis). Finally, in panel F., PC1 was plotted (on the X axis) and compared to PC4 (on the Y axis).

Supplementary Figure 2. The first four EIGENSTRAT principal component analyses plots showing the distribution of presumed half-siblings (based on the genomic relationship matrix) of the ten sires with the most offspring within the New Mexico calf population. Each sire's offspring are coded with a different color and tend to cluster together within the study population. In panel A., the first principal component (PC1) (plotted on the X axis) is compared against the second principal component (PC2)(on the Y axis). In panel B., PC1 is again plotted on the X axis but is now compared against the third principal component (PC3) on the Y axis. Principal component 2 (PC2) (on the X axis) is plotted against the third principal component (PC3)(on the Y axis) in panel C. In Panel D., PC1 (on the X axis) was plotted against principal

component 4 (PC4) (on the Y axis). In panel E., PC2 (on the X axis) was compared to PC4 (on the Y axis). Finally, in panel F., PC1 was plotted (on the X axis) and compared to PC4 (on the Y axis).

Supplementary Figure 3. EIGENSTRAT Q-Q plots: A. CA with correction of 100 principle components and age and sex included in the model, B. NM calves with correction of 5 principle components with only age included in the model, and C. the combined CA-NM population with correction of 80 principle components and sex and age included in the model. For each of the plots in panels A-C, the observed –log10 *P*-value (on the Y axis) is plotted against the expected –log10 *P*-value (on the X axis).

Supplementary Figure 4. EMMAX-GRM P-P plots with age and sex in the model for: A. California,and B. NM. In panel C., sex, age and population of origin were included in the combined CA-NM population. For each of the plots in panels A-C, the observed –log10 *P*-value (on the Y axis) is plotted against the expected –log10 *P*-value (on the X axis).

Supplementary Figure 5. FvR P-P plots with sex and age in the model for: A. California with correction for 53 principle components, and B. NM calves with correction of 9 principle components. In panel C. sex, age and population of origin were included in the combined CA-NM population which included correction of 91 principle components. . For each of the plots in panels A-C, the observed –log10 *P*-value (on the Y axis) is plotted against the expected –log10 *P*-value (on the X axis).

Note on Supplemental Q-Q and P-P Plots: The trend observed in the Q-Q and/or P-P plots whereby the expected –log10 *P*-values are larger than the observed was determined to be an issue related to the Holstein breed and/or the samples utilized. Briefly, LD pruning prior to GWAA mitigates this trend, which has never been observed during any of our ongoing QTL GWAA for beef cattle (i.e., multiple breeds, thousands of samples) using the same methods employed in this study. Unfortunately, very few Q-Q / P-P plots exist in the literure for U.S. Holstein cattle, and because we genotyped and analyzed ≥ 2,596 holstein calves, we are confident that this result will be replicated. Finally, the trend whereby the expected –log10 *P*-values are larger than the observed –log10 *P*-values is less noticeable when using a PCA-based approach to stratification control during a GWAA because, given the number of components used, these PCA methods are not as exhaustive at correcting for stratification, as is the genomic relationship matrix.

References for Additional File

1. McGuirk SM: **Disease management of dairy calves and heifers.** *Vet Clin North Am Food Anim Pract* 2008, **24:**139-153.

2. Brower A, Homb KM, Bochsler P, Porter R, Woods K, Ubl S, Krueger D, Cigel F, Toohey-Kurth K: **Encephalitis in aborted bovine fetuses associated with Bovine herpesvirus 1 infection.** *J Vet Diagn Invest* 2008, **20:**297-303.

3. Mahlum CE, Haugerud S, Shivers JL, Rossow KD, Goyal SM, Collins JE, Faaberg KS: **Detection of bovine viral diarrhea virus by TaqMan reverse treanscription polymerase chain reaction.** *J Vet Diagn Invest* 2002, **14:**120-125.

4. Letellier C, Boxus M, Rosar L, Toussaint JF, Walravens K, Roels S, Meyer G, Letesson JJ, Kerkhofs P: **Vaccination of calves using the BRSV nucleocapsid protein in a DNA prime–protein boost strategy stimulates cell-mediated immunity and protects the lungs against BRSV replication and pathology.** *Vaccine* 2008, **26:**4840-4848.

5. Kang HM, Sul JH, Service SK, Zaitlen NA, Kong SY, Freimer NB, Sabatti C, Eskin E: **Variance component model to account for sample structure in genome-wide association studies.** *Nat Genet* 2010, **42:**348-354.

6. Segura V, Vihjálmsson BJ, Platt A, Korte A, Seren Ü, Long Q, Nordborg M: **An efficient multi-locus mixed-model approach for genome-wide association studies in structured populations.** *Nat Genet* 2012, **44:**825–830.

7. VanRaden PM: **Efficient methods to compute genomic predictions.** *J Dairy Sci* 2008, **91:**4414-4423.

8. Pinedo PJ, Galvão KN, Seabury CM: **Innate immune gene variation and differential susceptibility to uterine diseases in Holstein cows.** *Theriogenology* 2013, **80**(4):384-390.

9. Purcell S, Neale B, Todd-Brown K, Thomas L, Ferreira MA, Bender D, Maller J, Sklar P, de Bakker PI, Daly MJ, Sham PC: **PLINK: a toolset for whole-genome association and population-based linkage analysis.** *Am J Hum Genet* 2007, **81:**559-575.

10. Price AL, Patterson NJ, Plenge RM, Weinblatt ME, Shadick NA, Reich D: **Principal components analysis corrects for stratification in genome-wide association studies.** *Nat Genet* 2006, **38:**904-909.

11. Taylor JF: **Implementation and accuracy of genomic selection**. *Aquaculture* 2014, 420-**421**(Suppl 1):8-14.

12. Browning BL, Browning SR: **A unified approach to genotype imputation and haplotype phase inference for large data sets of trios and unrelated individuals.** *Am J Hum Genet* 2009, **84:**210-223.
