# Supplementary figures and images for: Susceptibility loci revealed for bovine respiratory disease complex in pre-weaned holstein calves
Source: BMC Genomics. 2014 Dec 22;15(1):1164. doi: 10.1186/1471-2164-15-1164 (PMC4445561; doi:10.1186/1471-2164-15-1164)

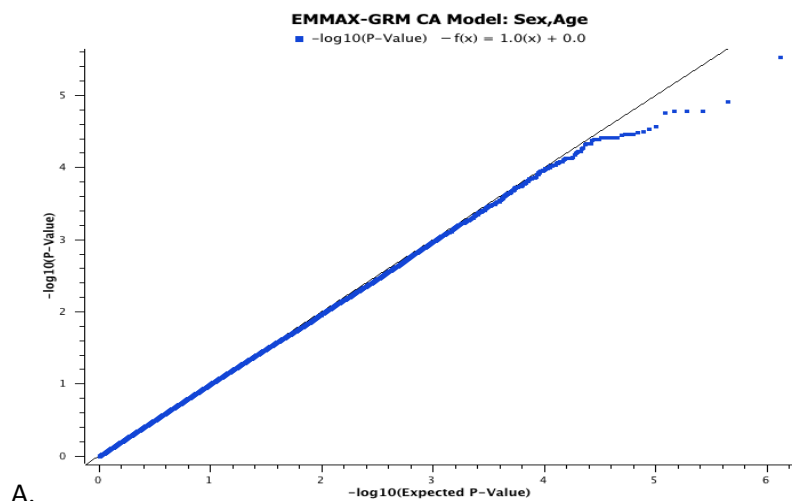

A.

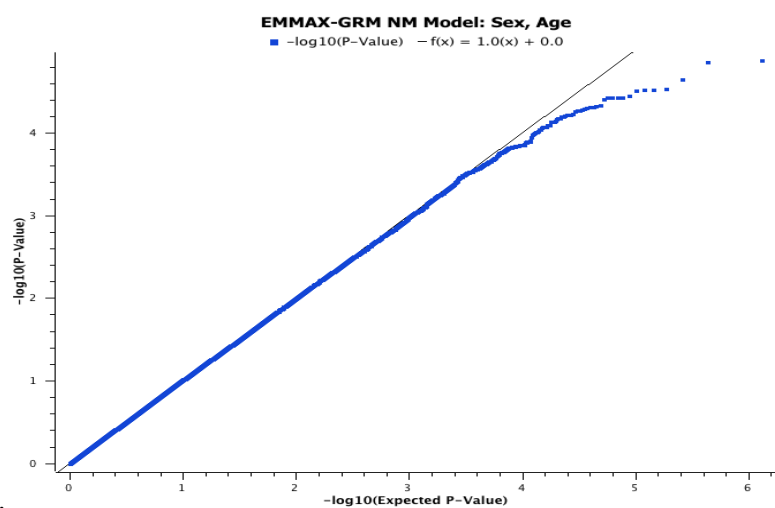

B.

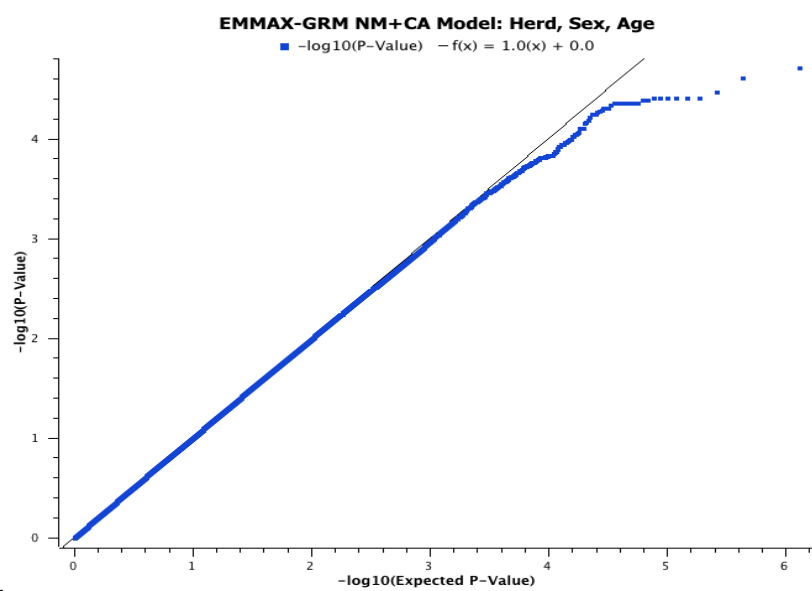

C.

Supplement: Supplementary file 2 — Additional file 2: Figure S1: EMMAX-GRM P-P plots with age and sex in the model for: A. California, and B. NM. In panel C., sex, age and population of origin were included in the combined CA-NM population. For each of the plots in panels A-C, the observed –log10 P-value (on the Y axis) is plotted against the expected –log10 P-value (on the X axis). (PDF 126 KB) [file 12864_2014_7079_MOESM2_ESM.pdf]

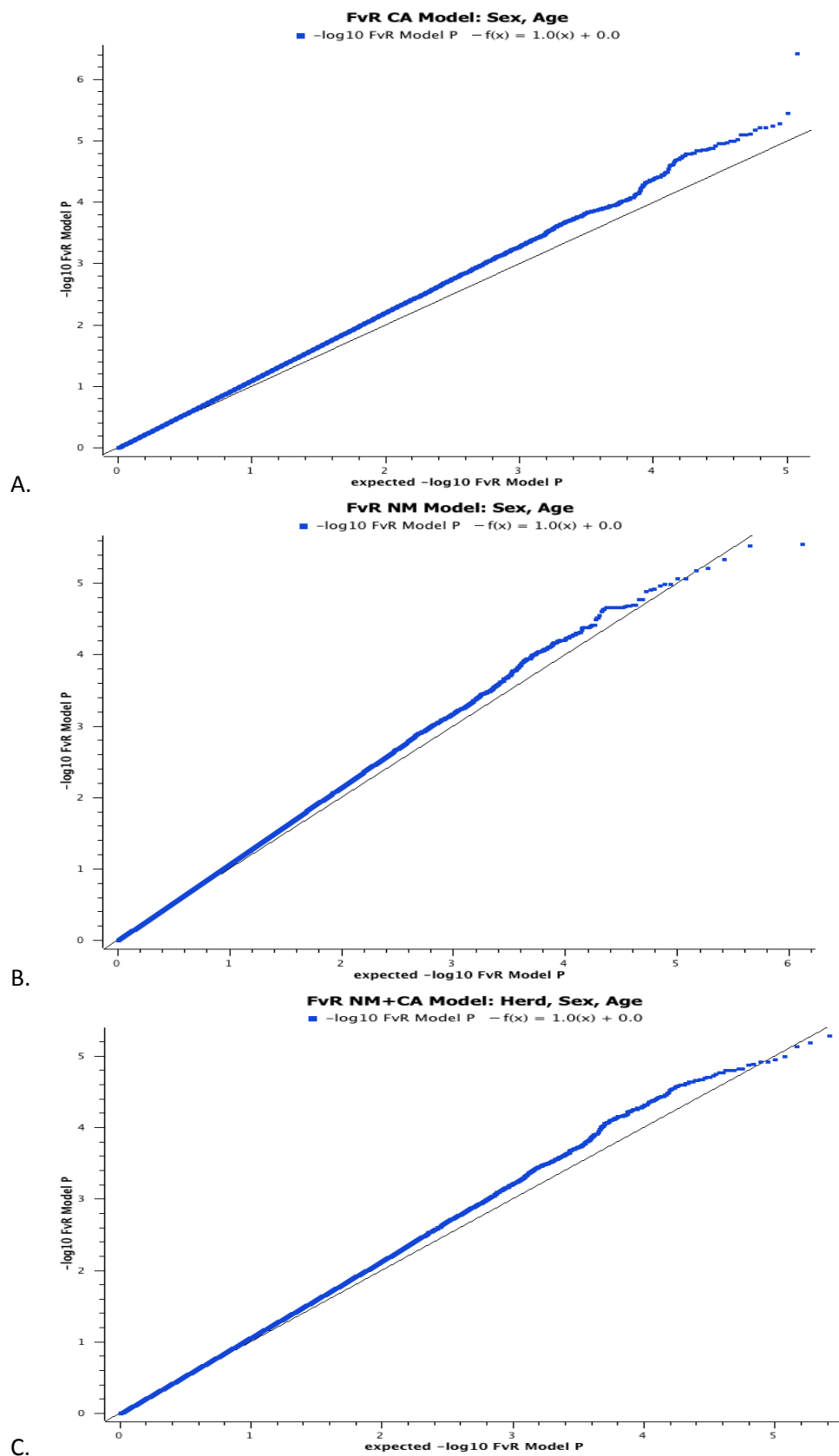

Supplement: Supplementary file 3 — Additional file 3: Figure S2: FvR P-P plots with sex and age in the model for: A. California with correction for 53 principle components, and B. NM calves with correction of 9 principle components. In panel C. sex, age and population of origin were included in the combined CA-NM population which included correction of 91 principle components. For each of the plots in panels A-C, the observed –log10 P-value (on the Y axis) is plotted against the expected –log10 P-value (on the X axis). (PDF 128 KB) [file 12864_2014_7079_MOESM3_ESM.pdf]

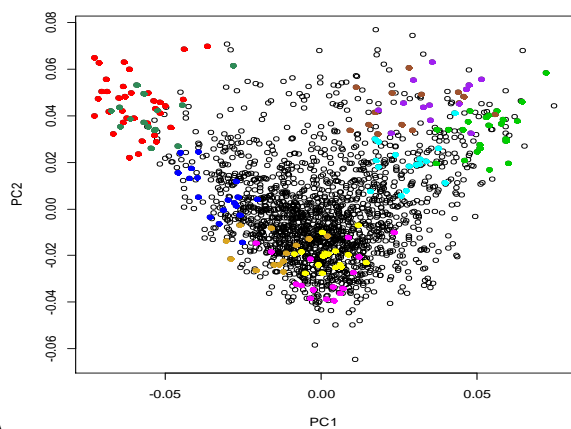

A

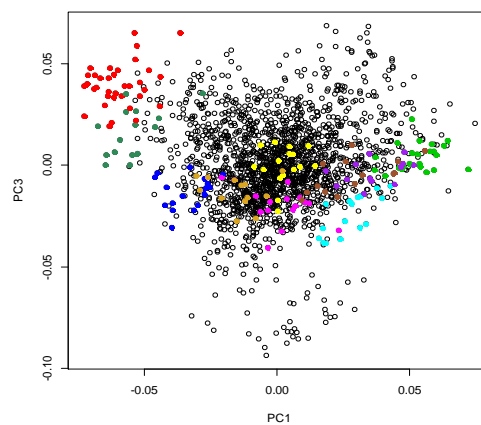

B.

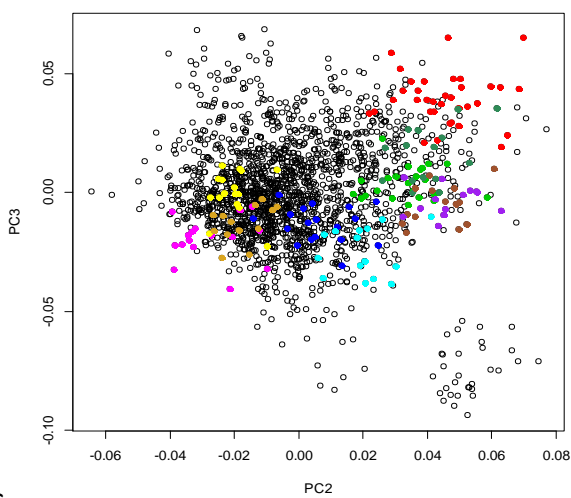

C

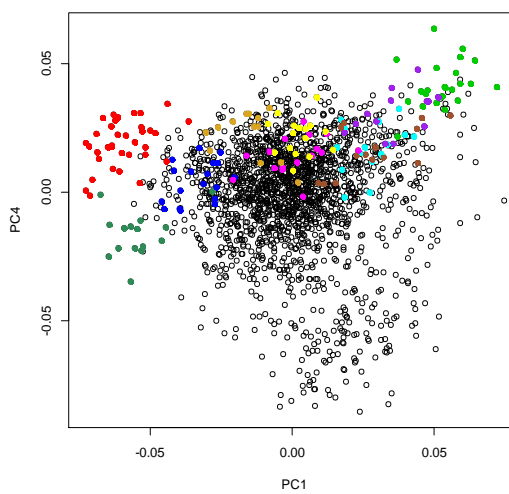

D.

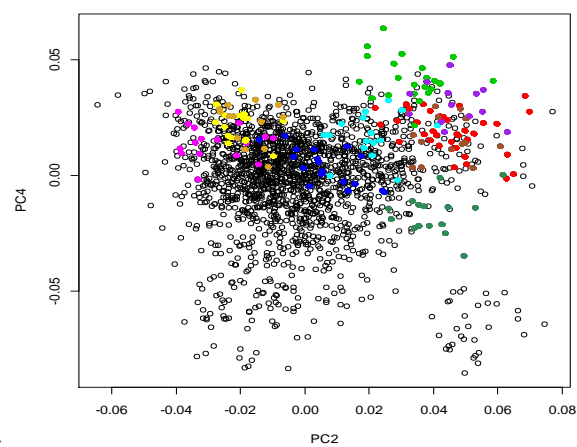

E.

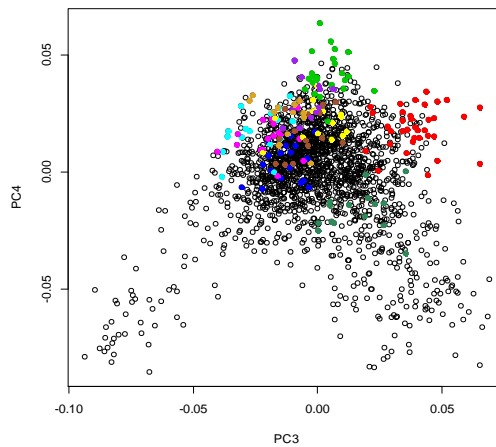

F.

Supplement: Supplementary file 4 — Additional file 4: Figure S3: The first four EIGENSTRAT principal component analyses plots showing the distribution of presumed half-siblings (based on the genomic relationship matrix) of ten sires with the most offspring within the California calf study population. Each sire’s offspring are coded with a different color and tend to cluster together within the study population. In panel A., the first principal component (PC1) (plotted on the X axis) is compared against the second principal component (PC2)( on the Y axis). In panel B., PC1 is again plotted on the X axis but is now compared against the third principal component (PC3) on the Y axis. Principal component 2 (PC2) (on the X axis) is plotted against the third principal component (PC3)( on the Y axis) in panel C. In Panel D., PC1 (on the X axis) was plotted against principal component 4 (PC4) (on the Y axis). In panel E., PC2 (on the X axis) was compared to PC4 (on the Y axis). Finally, in panel F., PC1 was plotted (on the X axis) and compared to PC4 (on the Y axis). (PDF 816 KB) [file 12864_2014_7079_MOESM4_ESM.pdf]

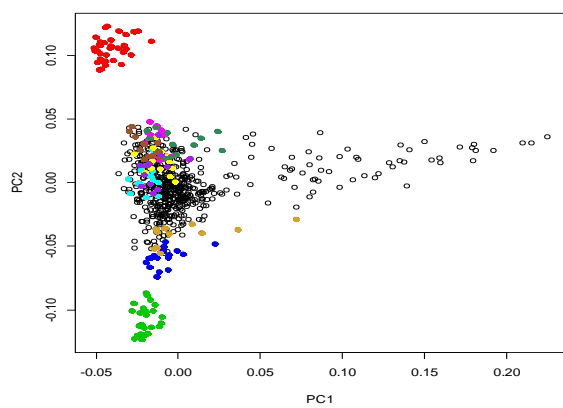

A.

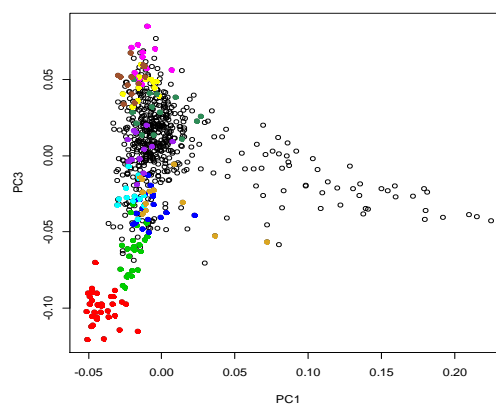

B.

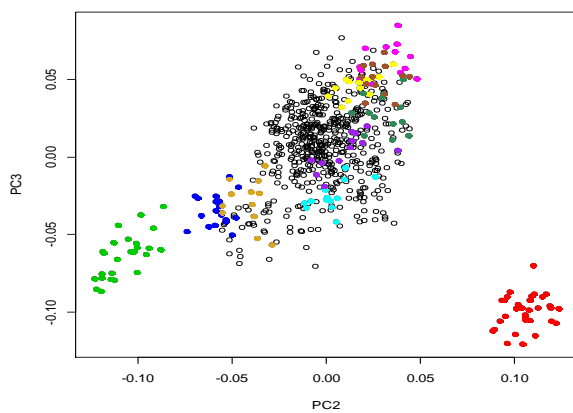

C.

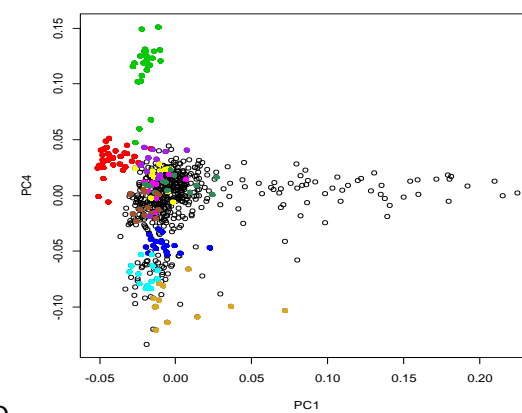

D.

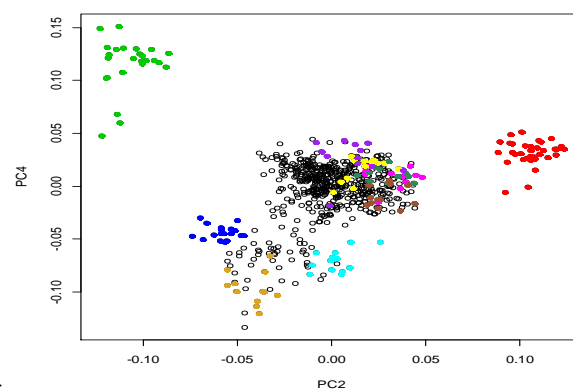

E.

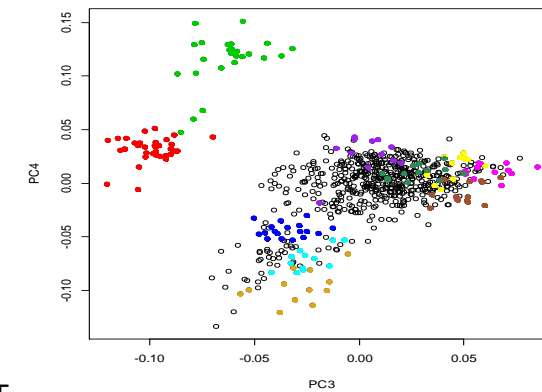

F.

Supplement: Supplementary file 5 — Additional file 5: Figure S4: The first four EIGENSTRAT principal component analyses plots showing the distribution of presumed half-siblings (based on the genomic relationship matrix) of the ten sires with the most offspring within the New Mexico calf population. Each sire’s offspring are coded with a different color and tend to cluster together within the study population. In panel A., the first principal component (PC1) (plotted on the X axis) is compared against the second principal component (PC2)( on the Y axis). In panel B., PC1 is again plotted on the X axis but is now compared against the third principal component (PC3) on the Y axis. Principal component 2 (PC2) (on the X axis) is plotted against the third principal component (PC3)( on the Y axis) in panel C. In Panel D., PC1 (on the X axis) was plotted against principal component 4 (PC4) (on the Y axis). In panel E., PC2 (on the X axis) was compared to PC4 (on the Y axis). Finally, in panel F., PC1 was plotted (on the X axis) and compared to PC4 (on the Y axis). (PDF 360 KB) [file 12864_2014_7079_MOESM5_ESM.pdf]

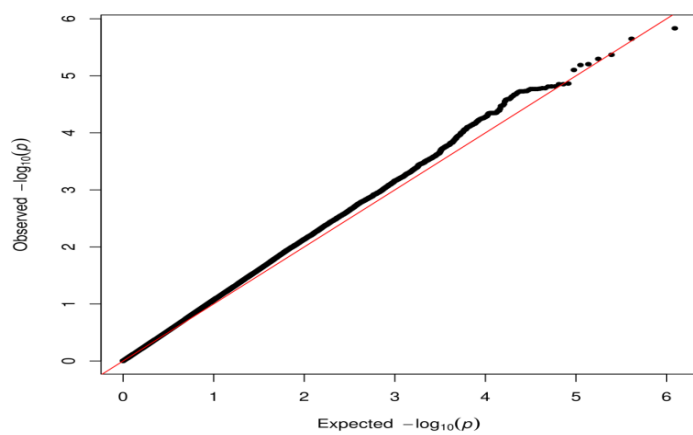

A.

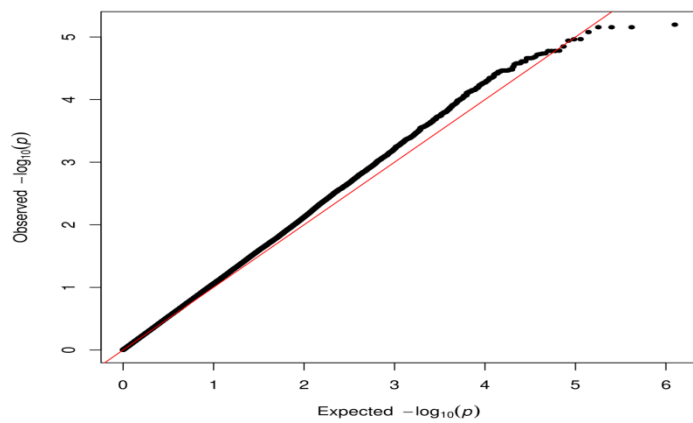

B.

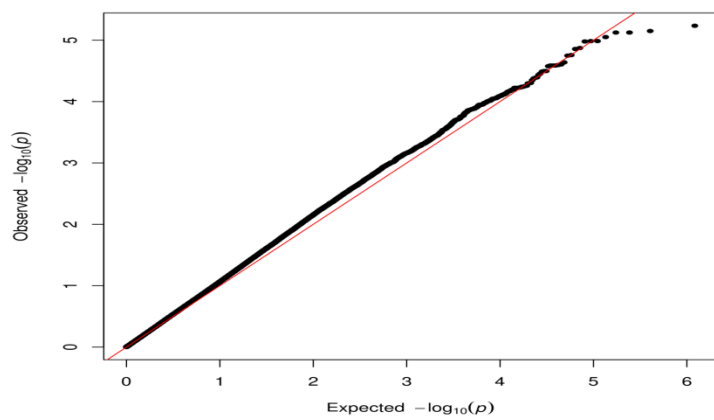

C.

Supplement: Supplementary file 6 — Additional file 6: Figure S5: EIGENSTRAT Q-Q plots: A. CA with correction of 100 principle components and age and sex included in the model, B. NM calves with correction of 5 principle components with only age included in the model, and C. the combined CA-NM population with correction of 80 principle components and sex and age included in the model. For each of the plots in panels A-C, the observed –log10 P-value (on the Y axis) is plotted against the expected –log10 P-value (on the X axis). (PDF 169 KB) [file 12864_2014_7079_MOESM6_ESM.pdf]
